# Secreted Aspartic Proteinases: Key Factors in *Candida* Infections and Host-Pathogen Interactions

**DOI:** 10.3390/ijms25094775

**Published:** 2024-04-27

**Authors:** Grazyna Bras, Dorota Satala, Magdalena Juszczak, Kamila Kulig, Ewelina Wronowska, Aneta Bednarek, Marcin Zawrotniak, Maria Rapala-Kozik, Justyna Karkowska-Kuleta

**Affiliations:** 1 Department of Comparative Biochemistry and Bioanalytics, Faculty of Biochemistry, Biophysics and Biotechnology, Jagiellonian University, Gronostajowa 7, 30-387 Kraków, Polandmarcin.zawrotniak@uj.edu.pl (M.Z.); justyna.karkowska@uj.edu.pl (J.K.-K.); 2 Doctoral School of Exact and Natural Sciences, Faculty of Biochemistry, Biophysics and Biotechnology, Jagiellonian University, Gronostajowa 7, 30-387 Kraków, Poland

**Keywords:** *Candida*, candidiasis, proteinases, Saps, proteolysis, protein degradation, virulence

## Abstract

Extracellular proteases are key factors contributing to the virulence of pathogenic fungi from the genus *Candida*. Their proteolytic activities are crucial for extracting nutrients from the external environment, degrading host defenses, and destabilizing the internal balance of the human organism. Currently, the enzymes most frequently described in this context are secreted aspartic proteases (Saps). This review comprehensively explores the multifaceted roles of Saps, highlighting their importance in biofilm formation, tissue invasion through the degradation of extracellular matrix proteins and components of the coagulation cascade, modulation of host immune responses via impairment of neutrophil and monocyte/macrophage functions, and their contribution to antifungal resistance. Additionally, the diagnostic challenges associated with *Candida* infections and the potential of Saps as biomarkers were discussed. Furthermore, we examined the prospects of developing vaccines based on Saps and the use of protease inhibitors as adjunctive therapies for candidiasis. Given the complex biology of Saps and their central role in *Candida* pathogenicity, a multidisciplinary approach may pave the way for innovative diagnostic strategies and open new opportunities for innovative clinical interventions against candidiasis.

## 1. *Candida* Pathogenic Yeasts—Their Clinical Relevance as Opportunistic Pathogens

*Candida* species are predominantly commensal microorganisms that establish colonization on the cutaneous and mucosal surfaces of a significant part of the human population [1,2]. Their proliferation and location are regulated by the efficient action of the host immune system. However, fungi from the genus *Candida* possess an extensive repertoire of virulence factors and pathogenicity mechanisms, which can be mobilized upon disruption of the fragile equilibrium among the endogenous microbiota, the immunological defense system of the host, and the *Candida* cells themselves, thereby facilitating their transition to opportunistic pathogens and further development of infection [3,4]. The highest risk of developing infections caused by *Candida* concerns individuals with a weakened immune system, either as a result of congenital immunodeficiency, diabetes, HIV infection, or as a consequence of surgical procedures, injuries, cancer, chemotherapy; also patients in long-term intensive care units, treated with glucocorticoids or long-term antibiotic therapy, parenteral nutrition, or low birth weight infants [5,6]. Disease states attributable to the representatives of the *Candida* genus encompass a spectrum ranging from relatively harmless but troublesome superficial infections to dangerous systemic infections such as candidemia or systemic candidiasis [3]. These life-threatening conditions currently pose a clinical challenge due to their diverse manifestations, severe morbidity, and endangering individuals affected by other serious medical conditions. One such infection of nosocomial emergence is invasive candidiasis, with a worldwide frequency of up to half a million individuals each year and mortality rates close to 55% [7,8]. While *C. albicans* still constitutes the most common cause of candidiasis worldwide, non-*albicans Candida* species are presently increasingly identified, and for *C. auris*, there has been an expansive increase in the incidence of infections over the last decade [9,10,11,12].

Amongst numerous multifaceted conditions that make representatives of the *Candida* genus such widespread opportunistic pathogens are their ability to plastically adapt to the prevailing environmental requirements and to trigger available physiological and pathological mechanisms to survive in the host organism [13]. A pivotal component of this adaptive arsenal is the production and secretion of different proteins that play critical roles in host–pathogen interactions. This group includes extracellular hydrolases that are involved not only in various physiological processes but also act as key contributors to the fungal virulence correlated with the candidal capability to invade the human host and spread the infection further [14,15]. To date, the enzymes most frequently described in this context are secreted aspartic proteases (Saps). The presence of these enzymes has been confirmed at the genetic level for several *Candida* species [16,17,18,19]. Proteinase-deficient mutants of *C. albicans* showed significantly reduced virulence, highlighting the role of these enzymes in infectious processes [20].

The most thoroughly studied representatives of this group of enzymes so far are *C. albicans* Sap1-10 [16,21], *C. parapsilosis* Sapp1, Sapp2 [19,22,23], *C. tropicalis* Sapt1-Sapt4 [24], *C. dubliniensis* CdSap1-4 and CdSap7-10 [25] and *C. auris* Sapa1-7 [26]. Proteinase Sap2 produced by *C. albicans* plays a crucial role in the fungal ability to proliferate in environments where protein is the sole nitrogen source, which has been empirically validated through the study of *sap2*Δ mutants, which exhibited impaired growth under these conditions [27,28], as well as in research using the *C. albicans* mutant strain deprived of Ecm33 protein, in which the secretion of Sap2 and consequently degradation of proteins in the culture medium were significantly altered [29]. Furthermore, with regard to the effect on the cell biology and virulence of yeasts, considerable importance has also been repeatedly assigned to extracellular glycosylphosphatidylinositol (GPI)-linked aspartic proteases—yapsins (NgYps1-11) [30]—produced by the species *Nakaseomyces glabratus*, which was previously included in the genus *Candida* as *C. glabrata* [31].

*C. albicans* Saps are produced as preproenzymes equipped with a signal peptide and a propeptide, which are removed during proteolytic processing of the polypeptide chain by subtilisin-like protease, proprotein convertase Kex2, to generate secreted active hydrolases [32]. A similar processing mechanism was also demonstrated for Saps produced by *C. tropicalis* [24,33] and *C. parapsilosis* [34,35]. Nevertheless, autocatalytic activation has also been reported for *C. albicans* and *C. parapsilosis* proteases [34,36]. Members of the Sap family contain four conserved cysteine residues responsible for the maintenance of three-dimensional structure and two conserved aspartate residues essential for proteolytic activity [37]. *C. albicans* Sap9 and Sap10, unlike the other members of this family, are equipped at their C-terminus with GPI anchors attaching them to the cell wall or the cell wall and plasma membrane [37]. While the canonical protein secretion pathway is primarily assigned to the extracellular transfer of Saps, it has also been demonstrated recently that these hydrolytic enzymes are also transported within extracellular vesicles (EVs) produced by the fungal cells [38,39,40,41,42].

Within particular families, individual enzymes differ in their physicochemical properties, substrate specificity, and optimal conditions for enzymatic activity [43,44,45]. A broad substrate specificity was reported for *C. albicans* Sap1–Sap6 and Sap8, contrary to Sap7, Sap9, and Sap10, characterized by narrower substrate specificity [36,37,46]. As for *C. parapsilosis*, Sapp2 exhibits a quite narrow substrate specificity and lower catalytic activity than Sapp1 [47]. Moreover, in the case of *C. albicans* Saps, pH within the range of 3.0–5.0 was indicated as optimal for their proteolytic activity [44]; however, these enzymes are also active at a pH close to neutral, which significantly expands their potential range of action [44,48]. *C. tropicalis* Sapt2 and Sapt3 had a pH optimum of 5.0, while the optimal pH for Sapt1 activity was assigned to 3.5 [24]. In *C. albicans*, both the Cph1-mediated MAP kinase cascade and the Efg1-mediated cAMP/PKA pathway are involved in the regulation of the expression of hypha-associated *SAP* genes [49]. For the regulation of the activity of *C. auris* Saps, including the major proteinase Sapa3, the importance of Ras/cAMP/PKA signaling pathway, with the crucial role of PKA catalytic subunits, has been demonstrated; however, further detailed studies are needed to elucidate thoroughly the factors influencing expression patterns and activity of this proteinase [26].

**Figure 1:**
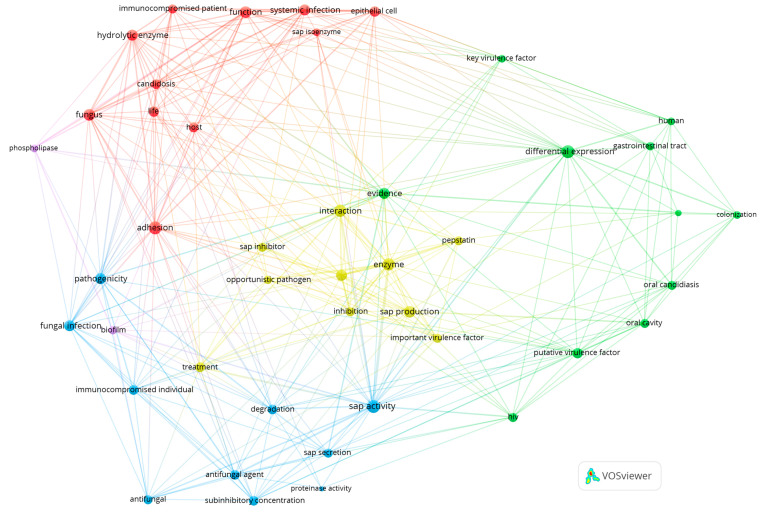
Network visualization of terms related to the relationship between *Candida* Saps and virulence, documented in the scientific literature in the years 1993–2023 based on the Web of Science database (VOSviewer version 1.6.19, Centre for Science and Technology Studies, Leiden University, The Netherlands). Terms that co-occur are located close to each other in the visualization, and related terms are grouped into five clusters, indicated with distinct colors.

The construction of a network of links between terms, performed with VOSviewer [50] and based on bibliographic data from the Web of Science Core Collection database from 1993 to 2023 with a search query for topics ‘*Candida*’, ‘Sap’, and ‘virulence’, showed the noticeable presence of relations between these terms in the published scientific literature, referring to the involvement of these fungal enzymes as key virulence factors in the different pathological processes, including adhesion, biofilm formation, protein degradation, invasion to host tissues, and contribution to antifungal resistance (Figure 1).

As Saps and yapsins are significantly involved in numerous aspects of host–pathogen interactions, from the degradation of host proteins to the evasion of immune responses, there remain considerable gaps in our understanding of their functionality and potential in the pathogenesis of *Candida* infections. Therefore, this review aims to consolidate current knowledge about this group of fungal proteolytic enzymes and explore the usability of *Candida* aspartic proteases as targets for novel antifungal therapies, markers important for the diagnosis of candidiasis, and promisingly effective vaccine components.

## 2. Various Pathophysiological Functions of SAPs

### 2.1. Biofilm Formation

A distinctive characteristic of the pathogenicity of *Candida* species is their ability to develop biofilms. These structures are highly resistant to antimicrobial treatments and are linked to persistent host colonization and infections. Biofilms consist of various morphological forms of fungal cells that adhere to artificial surfaces or living tissues. They produce extracellular polymers, creating a protective matrix that shields them from external influences, including the host’s immune defenses and the effects of antifungal drugs [51,52]. A comparison of the metabolic activity between planktonic yeast cells and those composing biofilms has documented an upregulation of protease activities in the latter [53].

The formation of *C. albicans* biofilm is a complex, multistep process that involves the adsorption and adhesion of yeast cells to the appropriate surface, accompanied by morphological changes in the cells; the formation of microcolonies and the production of an extracellular matrix, biofilm maturation, and finally, the dispersion of new yeast cells from the mature biofilm, which acts as a reservoir for recurrent infections [54].

Investigating the involvement of aspartic protease in biofilm formation encompasses the analysis of the expression of genes encoding these enzymes, observation of biofilm formation capabilities by corresponding deletion strains, and examination of biofilm development across various infection models. Genome-wide transcriptional analysis of *C. albicans* cells during biofilm formation has revealed that nearly all genes encoding secreted aspartyl proteases were significantly upregulated. However, the preference for specific stages of development or the infection model used varied [55]. For example, preferential upregulation of *SAP1* expression was identified in in vitro models, whereas *SAP2*, *SAP4*, and *SAP6* expression was detected in both in vivo and in vitro biofilm models [56]. Similarly, upregulation of the *SAP9* and *SAP10* genes, which encode proteinases associated with the fungal cell wall, was observed in cells forming biofilms. These proteinases play a critical role in maintaining the integrity of the fungal cell wall [46,57].

Saps in biofilm leverage their proteolytic properties to modify host or fungal cell surfaces, thereby exposing proteins more conducive for fungal adhesion. Interestingly, Saps can also act independently of their enzymatic activity, serving as binding ligands [58,59]. The adhesion capabilities of Sap proteins are attributed to RGD/KGD motifs found within their sequences. Notably, Sap4 contains an RGD motif, Sap5 includes an RGDKGD sequence, and Sap6 features two adjacent RGD motifs. These specified sequences, located near the enzyme’s active site cavity, are recognized by oral epithelial cell integrins. This recognition facilitates the adhesion to host tissue—a crucial step initiating biofilm formation—or leads to the internalization of the protease, resulting in proteolytic apoptotic outcomes for host cells [60]. Additionally, the sequences in Sap6 play a pivotal role in the self-aggregation of *C. albicans* cells, promoting fungal cell adhesion and biofilm formation. Furthermore, the identification of four amyloid-forming regions in Sap6, which are supported by the binding of zinc ions, significantly enhances the formation of fungal plaques by germinating cells [61].

Comparison of biofilm development between the wild-type and deletion mutant strains under dynamic flow conditions revealed a marked reduction in biofilm thickness for the mutant strains *sap5*Δ/Δ and *sap6*Δ/Δ, with *sap6*Δ/Δ exhibiting a more pronounced effect. This finding suggests that both proteases, which are pivotal for hyphal structures that infiltrate host tissue, play essential roles in the development and maturation of the *C. albicans* biofilm. Notably, Sap6 has emerged as a “community-organizing” molecule, underscoring its significant influence on biofilm architecture and function [53]. These findings are further corroborated by the observed increase in the expression of *SAP5* and *SAP6* genes during biofilm formation, underscoring the critical need for these proteases in the biofilm maturation process. Beyond their primary roles, these enzymes may serve additional functions crucial for biofilm development, such as facilitating nutrient acquisition, enhancing cell-to-cell communication, or contributing to the production of the extracellular matrix [56,62].

On the other hand, the expression of *SAP9* and *SAP10* within biofilms is constitutive, exhibiting relatively similar levels across various biofilm models [56]. The principal function attributed to these proteases within biofilms is the proteolytic modification of proteins exposed on the cell surface. Notably, the cell-associated chitinase Cht2 was identified as one such protein whose activity is significantly diminished in mutant strains lacking the *SAP9* and *SAP10* genes. This finding suggests a direct link between the proteolytic processing of Cht2 and its role in the remodeling of the cell wall [46]. Furthermore, the rearrangement of cell wall components is exemplified by the modification in Pir1’s structure, which, facilitated by both proteases, leads to an alteration in its cross-linking to the cell surface [46]. A more intricate function of Sap9 is observed within mixed-species biofilms, where it acts as a structural modulator for the primary fungal adhesins, Epa1 and Als3. These adhesins are crucial for the initial binding of fungal cells to artificial surfaces or host tissues, thus playing a key role in the formation of early-stage biofilms [63,64,65]. Both adhesins, which exhibit a preference for hyphal locations, also contain functional amyloid-forming sequences that contribute to cell–cell aggregation. This phenomenon is particularly evident in the biofilm formed by the *C. albicans sap9*Δ mutant strain [66]. The observed alterations in the biofilm structure, facilitated by cells expressing Sap9, suggest that this protease may play a role in the trimming of adhesins and the disaggregation of hyphal filaments. Such processes potentially enhance the hyphae’s ability to penetrate the environment more effectively. Furthermore, Sap9 may also act as a cooperative factor in these interactions, either through direct binding to Epa1 or by processing the cell surface proteins that are recognized by Eap1 [67]. 

Furthermore, Sap9 is implicated in interkingdom interactions, wherein the proteolytic modification of fungal adhesins may govern the composition of mixed-species biofilm [64,67,68,69]. The proteolytic activity of Sap9 might also contribute to enhancing the competitive advantage of *C. albicans* in proliferating and persisting within oral microbiome communities. This enhancement is evidenced by proteolytic cleavage and subsequent elimination of pellicle binding sites for streptococci, as documented by Dutton et al. [67].

The final stage of biofilm maturation involves the dispersion of yeast cells that exhibit increased virulence and adhesion capabilities. These properties facilitate the formation of a new, more hazardous, and drug-resistant biofilm [70,71]. The heightened virulence traits of the biofilm dispersal cells have been linked to the upregulated expression of genes encoding proteases *SAP3*, *SAP6*, *SAP8*, and *SAP9* [72].

The multifaceted roles and activities of aspartic proteases throughout the various phases of fungal biofilm formation and the progression of infection underscore their potential as promising targets for specialized antibiofilm therapies. Efforts in this direction have already been demonstrated with the application of HIV aspartyl protease inhibitors, which have shown efficacy in curbing the development of *C. albicans* biofilms [73,74]. Similarly, the use of mycogenic Ag nanoparticles has been identified as effective in inhibiting biofilm development, indicating the potential for broad-spectrum applications in combating fungal infections [75]. Moreover, integrating such specific inhibitors with conventional antifungal agents, like amphotericin B or caspofungin, may enhance strategies for the prevention and treatment of *C. albicans* biofilm-associated infections [76].

### 2.2. Tissue Invasion and Damage

#### 2.2.1. Degradation of Host Barriers

The degradation of the host structures constituting the first line of physical barriers against invading pathogens is a critical prerequisite for the initiation of infection and its subsequent propagation. This process requires the degradation of various proteins with structural functions and has been demonstrated repeatedly. One example is the degradation of mucins—glycoproteins that are the key constituents of the mucus layer—described for *C. albicans* Sap2 [77,78]. In addition, *C. albicans* Sap5 can degrade E-cadherin, the major constituent of epithelial adherens junctions, which play a crucial role in preserving the structural integrity of the epithelial cell barrier [79].

The secretion of Saps to the external environment contributes also to the degradation of the tight network of extracellular matrix (ECM) proteins, which is the natural barrier that the pathogen must overcome to enter the bloodstream and cause systemic infection [80]. The spread of *C. albicans* through the circulatory system is a key step in systemic infection. The proteolytic activity of Saps, especially Sap2, may facilitate this process, facilitating yeasts’ access to the internal organs by degrading essential components of the ECM network. Sap2 was particularly specified as the most important candidal proteinase contributing to the degradation of ECM proteins, also due to its broad substrate specificity [81]. In 1990, the ability of the *Candida* protease to degrade several ECM proteins, including collagen, was confirmed [82], while a few years later, Morschhauser et al. studied *C. albicans* cells directly for their ability to degrade ECM components [83]. Radioactively labeled ECM was treated with *C. albicans* cells cultured to secrete proteinase Sap2 and the level of degradation of ECM components was analyzed by measuring the radioactivity released into the supernatant. It was demonstrated that the proteinases produced by yeast effectively degraded ECM; moreover, the addition of pepstatin A, an inhibitor of aspartic proteinases, caused a significant decrease in the measured signal, even comparable to the control without yeast cells [83]. Also, the degradation of two proteins, fibronectin and laminin, was shown [83], and these two human proteins are not only important structural ECM components, but also have key regulatory functions in intercellular communication, and their presence is essential for the proper functioning of tissues and the organism [84].

#### 2.2.2. Proteolysis of Proteins from Coagulation Cascade, Contact System, and Inhibitors of Plasma Proteinases

Disturbance of homeostasis of host proteolytic systems during bacterial and fungal infections is considered one of the mechanisms of their pathogenesis. Proteinases often play a crucial role in this phenomenon.

**Figure 2:**
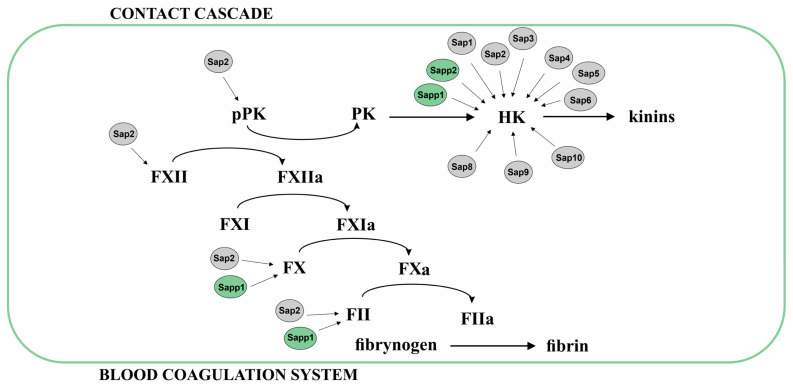
The influence of Sap proteases on the components of the plasma blood coagulation cascades. FII—coagulation factor II; FIIa—activated coagulation factor II; FX—coagulation factor X; FXa—activated coagulation factor X; FXI—coagulation factor XI; FXIa—activated coagulation factor XI; FXII—coagulation factor XII; FXIIa—activated coagulation factor XII; HK—high-molecular-weight kininogen; pPK—plasma prekallikrein; PK—plasma kallikrein.

Limited proteolysis of the blood coagulation cascade proteins—zymogens of serine proteinases, leading to the activation of these components and related with the action of microbial proteinases, may be responsible for septic clotting, insufficient peripheral circulation, disseminated intravascular coagulation during infection and, consequently, multi-organ failure [85]. The involvement of Saps in the activation of several coagulation factors has been demonstrated (Figure 2). Using normal human plasma, human plasmas deficient in factor XII (FXII) or factor X (FX), and purified clotting factors, the activation of FXII, FX, and factor II (prothrombin, FII) by *C. albicans* Sap2 was confirmed [85,86,87]. In the case of proteases of other *Candida* species, participation in clot formation has been proven only for *C. parapsilosis* Sapp1. This enzyme activates FXII and FII, which has also been demonstrated in in vitro studies [35,47]. In addition, the active form of FXII (FXIIa), generated by fungal proteases, can activate further molecules of FXII (autoactivation) and factor FXI, consequently stimulating clot formation indirectly.

FXII is a component not only of the coagulation system but also of the contact cascade, also known as the kallikrein/kinin system. Its active form, FXIIa, converts plasma prekallikrein (pPK) to plasma kallikrein (PK), which then digests the non-enzymatic component of this pathway—high-molecular-weight kininogen (HK)—generating multifunctional active peptides—kinins [88]. Kinins have, among others, vasoactive and proinflammatory properties, and their overproduction during infection may lead to the establishment of an environment favorable to the pathogen and the spread of infection [89]. In addition to FXII activation, Kaminishi et al. demonstrated Sap2-mediated conversion of pPK to PK [90]. Importantly, the consequence of pPK activation by fungal proteases is also subsequent PK-mediated FXII activation. The increase in vascular permeability related to the presence of kinins after injection of Sap2 proteinase to the dorsal skin of guinea pigs has been observed, while no increase was detected when Sap2 was inactivated by heating or pepstatin A [90]. Inhibition of PK formation from pPK has also been shown in vitro in the presence of known PK inhibitors, including corn trypsin inhibitor, inhibitor of FXIIa, and soybean trypsin inhibitor [90]. Direct production of kinins from HK and its tissue form—low-molecular-weight kininogen (LK)—was also reported, with the participation of the mixture of proteinases released to the culture media by several *Candida* species, especially *C. albicans* and C. *parapsilosis* [91]. In subsequent studies, the contribution of individual fungal enzymes in this process was characterized in detail. Generation of Met-Lys-bradykinin (MK-BK) by *C. albicans* Sap2 [92], MK-BK and Leu-Met-Lys-bradykinin (LMK-BK) by *C. parapsilosis* Sapp1 and Sapp2, especially from LK, was observed [93]. Although these peptides were not produced as effectively as kinins generated by PK and tissue kallikreins (TK), they have been shown to be able to interact with and activate kinin receptors in human cells [92,93]. It has also been shown that in human plasma, they are rapidly transformed into more active forms by host kininases [93]. Subsequently, the production of kinins by all recombinant *C. albicans* Saps, except for Sap7, was also reported [94]. The MK-BK peptide has been shown to be released from kininogens at the highest yield by Sap3 and the combination of Sap9 with any of the other Saps. This finding became the basis for the hypothesis of cooperative degradation of kininogens by several Saps, used by *C. albicans* to produce the most advantageous amount of kinins at infection sites [94]. 

Several protease inhibitors are present in host plasma to regulate host homeostatic pathways, proteolytic cascades, and proinflammatory responses, preventing their uncontrolled activation. It has been reported that microbial proteases, including Saps, can inactivate these inhibitory proteins, resulting in host tissue damage and pathogen dissemination. Kaminishi et al. showed that α2-macroglobulin and α1-protease inhibitor (α1PI)—inhibitors of many host serine proteases, e.g., thrombin, kallikrein, and plasmin—are degraded by Sap2 [87]. This explained the earlier observation that activation of coagulation factors and plasma thrombin production by Sap2 were not affected by these endogenous plasma protease inhibitors [85]. The inactivation of α1PI, which is also a regulator of action of neutrophil elastase (NE) associated with neutrophil extracellular traps (NET), was also confirmed for recombinant Sap1-Sap4 and Sap9, indicating the major cleavage site in the C-terminal part of α1PI, close to the reactive-site loop [95]. Impaired inhibition of NE has been shown to cause NET-dependent damage to epithelial and endothelial cells and increases their susceptibility to *Candida* colonization [95]. Sap-modified α1PI not only has a reduced ability to inhibit NE but also to bind IL-8 [95]. Another important inhibitor—human epidermal cysteine proteinase inhibitor cystatin A—is located in the upper epidermal layer and is one of the host inhibitors that have a defense function against exogenous pathogens. Tsushima et al. demonstrated that cystatin A is cleaved by Sap2 into small peptides deprived of inhibitory activity [96].

In addition to being kinin precursors, HK and LK are also inhibitors of cysteine proteinases. They inhibit host enzymes such as papain, cathepsins L, S, H, K, and B [97,98], and calpain [99]. The significant level of HK and LK degradation mediated by Saps could also suggest disruption of the inhibitory functions of kininogen; however, this issue needs further investigation [91,92,93].

### 2.3. Immune Evasion and Modulation

**Figure 3:**
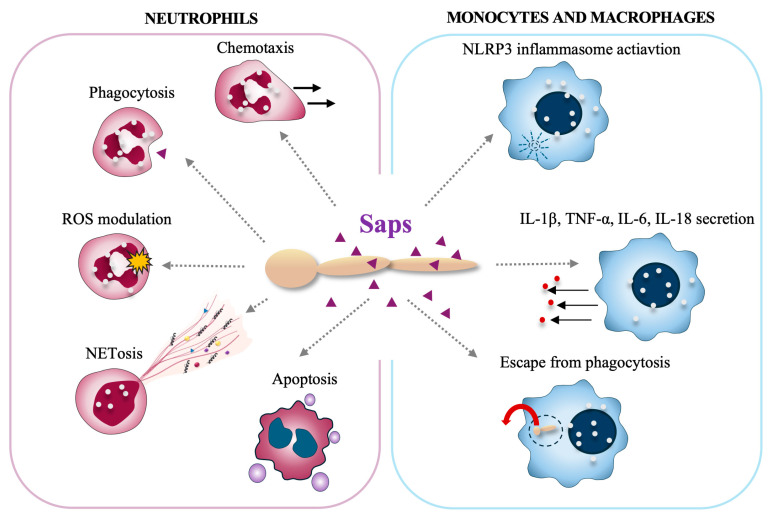
The influence of Sap proteases on the immune response of neutrophils (**left** panel) and monocytes and macrophages (**right** panel).

Proteinases produced by *Candida* also contribute significantly to the modulation of the response of the host immune system, including influencing the functionality of cells that are actively engaged in combating pathogens (Figure 3).

#### 2.3.1. Interactions with Neutrophils

Neutrophils represent the first nonspecific line of defense against pathogens, such as bacteria, fungi, and viruses, and are capable of neutralizing threats in the form of toxins or other potentially dangerous compounds [100]. They can fulfill their functions through a series of surface receptors that allow for the identification of foreign molecules, resulting in the activation of one of several defense mechanisms, such as phagocytosis [101], degranulation [102], and the release of neutrophil extracellular traps (NETs) [103]. Each of these mechanisms limits the spread of infection in a different way. Additionally, the ability of chemotaxis towards the site of inflammation significantly accelerates the cellular response.

Neutrophils are capable of recognizing the presence of *C. albicans* in host tissues [104], particularly the quorum-sensing molecules [105] and Saps released by the yeast [106]. The proteases Sap2 and Sap6 exhibit chemotactic action on neutrophils both in vitro (independently of the proteolytic activity of the enzymes) and indirectly in vivo through the induction of chemokines in the epithelium [107]. Additionally, the surface-bound Sap9 is directly responsible for neutrophil migration towards the filamentous form of *C. albicans*, independently of IL-8 production by neutrophils [108]. Sap9 also plays significant role in the recognition of the yeast by neutrophils, as well as in modulating the cells’ killing response. It has been shown that the survival of yeast with *SAP9* deletion was greater than that of the wild type in contact with neutrophils. This seems to be related to the activation of the oxidative burst mechanism in neutrophils, as demonstrated, the production of reactive oxygen species (ROS) was significantly lower, and the intensity of the oxidative burst was reduced in response to the *sap9*Δ strain of *C. albicans*. This proves the significant role of Sap9 in activating neutrophil defense mechanisms in response to the yeast. However, in contrast, the induction of apoptosis in neutrophils after two hours of contact with *C. albicans sap9*Δ strain was significantly lower than with wild-type cells. This was likely related to the reduced production of ROS in response to mutants, which are involved in the apoptotic signaling pathway [108].

Saps also play a significant role in activating the mechanism of NET release (netosis) [106]. The strongest response was observed for Sap4, Sap6, as well as Sap9 and Sap10, which are dominant for the filamentous form of *C. albicans*, that is more virulent than the unicellular yeast form and induces a stronger NET response upon contact with neutrophils [106]. The markedly lower response of neutrophils to Sap1-Sap3 treatments corresponded to their predominant expression in the yeast form of *C. albicans* [106,109], which is a less active inducer of netosis. The lowest potency found for Sap7 in terms of NET induction could be explained by its lowest sequence similarity to other members of the Sap family and its different structural properties [110]. Moreover, the *SAP7* gene is often not expressed during infection, and its role as a virulence factor has not been well established [109,111]. Sap1, Sap2, Sap8, Sap9, and Sap10 seem to activate a ROS-dependent pathway of netosis, as the presence of an NADPH oxidase inhibitor blocks this response mechanism. However, even in the presence of the inhibitor, partial release of NETs occurs in response to Sap4 and Sap6, suggesting that these two proteases may activate a ROS-independent pathway of netosis [106]. As shown, both Sap4 and Sap6 interact with the surface of neutrophils through CD11b (Mac-1) and CD11a (LFA-1) receptors. Moreover, Sap6 was also found to stimulate the CD14 receptor. In turn, the activation of netosis by Sap9 and Sap10 occurs through the action of CD16 (FcγRIIIB) and CD18 receptors [106]. All these receptors participate as initiators in the downstream activation of the Src/Syk kinase family [112,113], indicating the activation by Sap of a common netosis signaling pathway, also involving PI3K and ERK1/2 kinases.

Studies have shown that Sap6 interacts with the surface of neutrophils through Mac-1, leading to the internalization of the protease by immune cells [106]. This interaction occurs through the RDG-sequence motif, present in Sap6, as well as in Sap4 and Sap5, also involved in interactions with integrin on epithelial cells [60]. The process of binding to the surface and subsequent internalization seems crucial for regulating the neutrophil response to *C. albicans* and relates to ROS production or for the induction of epithelial cell apoptosis via a “Trojan horse” mechanism [60].

#### 2.3.2. Interactions with Monocytes/Macrophages

Monocytes and monocyte-derived macrophages are other key cells involved in fighting fungal infections. Monocytes and macrophages employ a diverse class of surface pattern-recognition receptors (PRRs), such as C-lectin receptors (CLRs), toll-like receptors (TLRs), and intracellular receptors, e.g., from the NOD-like family (NLR), to identify and engage *Candida* cells [114]. The binding of fungal antigens triggers the signaling pathways, such as the Akt/NF-κB pathway, that activate the direct effects, mainly cytokine production [115,116,117]. It has been shown that members of the Saps family exhibit varying capacities to stimulate the release of proinflammatory cytokines. Sap1, Sap2, and Sap6 significantly affect interleukin-1β (IL-1β), tumor necrosis factor alpha (TNF-α), and IL-6 secretion [116]. Additionally, in the case of Sap2 and Sap6, an increase in IL-18 production was observed. Notably, the observed IL-1β and IL-18 production associated with Sap2 and Sap6 is linked to the assembly of the NLRP3 inflammasome. The NLRP3 inflammasome is a critical multiprotein component of the innate immune system that mediates the activation of caspase 1 and proteolytic cleavage of pro-interleukin-1β (pro-IL-1β) and pro-IL-18 into their bioactive forms [117]. The initiation of NLRP3 inflammasome assembly requires prior internalization of Saps through a clathrin-dependent mechanism, intracellular induction of potassium efflux, and reactive oxygen production [117]. Studies indicate that activation of the NLRP3 inflammasome is critical for host defense against fungal infections because mice lacking NLPR3 exhibit greater susceptibility to *Candida* infections, resulting in increased mortality [118,119]. Subsequent research by Gabrielli et al. conducted on murine macrophages unveiled an additional noncanonical inflammasome activation pathway induced by Sap2 and Sap6 [120]. These findings indicated that Sap2 and Sap6 stimulate the production of substantial levels of type I interferon (IFN-α), which in turn affects caspase-11 activation. Caspase-11, in turn, is involved in the activation of caspase-1 and the subsequent production of IL-1β and IL-18 [120]. Studies on the immune response activated by *C. parapsilosis* have shown that mutants deprived of Sapp1/2/3 induce much lower production of proinflammatory cytokines (mainly IL-1β and IL-6) than reference strains, which highlights the role of these proteases in activating the macrophage response [19].

*Candida* species have also developed intricate strategies to target macrophages, enabling them to evade immune surveillance. In the study conducted by Borg-von Zepelin et al., the upregulation of Sap4 and Sap6 expression was noted following *Candida* phagocytosis by murine peritoneal macrophages [121]. Notably, *C. albicans* mutants deficient in Sap4 and Sap6 exhibit increased susceptibility to intracellular killing [121]. In the case of studies on *C. parapsilosis*, it was shown that mutants lacking Sapp1 (Δ/Δ*sapp1a* Δ/Δ*sapp1b*) were shown to be phagocytosed and killed more efficiently by human monocytes and macrophages than the cells of the wild-type strain [19]. These observations strongly imply the potential involvement of certain aspartic proteases, mainly Sap4, Sap6, and Sapp1, in the evasion of host immune defenses, but more detailed research is needed.

#### 2.3.3. Proteolysis of Complement, Antibodies, and Antimicrobial Peptides

The proteolytic function of aspartic proteases may also support fungal pathogens in evading host immune responses through direct action on plasma defense proteins. Cleavage of molecules such as immunoglobulins, antimicrobial peptides (AMPs), or complement system proteins may promote their inactivation and modulation of the host response. 

Antimicrobial peptides (AMPs) produced by the host in response to the presence of yeasts are considered a first-line defense against the progression of the infection. One strategy to avoid the antimicrobial effects of AMPs is the secretion of aspartic proteases. It has been shown that Sap1–Sap4 and Sap7–Sap10 [122,123] can cleave the antifungal peptide histatin-5 (Hst5) found in human saliva [122]. However, shorter derivatives of this peptide formed during the initial stages of hydrolysis—Hst5 1–21, 1–17, and 1–13—retain their activity, which may contribute to delaying the loss of the antimicrobial function of Hst5 [123]. Recently, it has been shown that introducing a change in the amino acid sequence of Hst5 (K11R and K17R) can improve the antifungal ability of this peptide and lead to the acquisition of proteolytic resistance [124,125] and further increase the ability to inhibit the formation of *C. albicans* biofilms [126].

Another antifungal peptide exhibiting additional immunomodulatory properties is human cathelicidin LL-37, which is produced constitutively in neutrophils and human epithelial cells [127]. Analogously to the Hst5 peptide, LL-37 can also be cleaved by Saps, specifically by Sap1–Sap4 and Sap8–Sap9. Two shorter derivative peptides—LL25 and LL8-37—are formed at the initial stages of the proteolytic degradation process, which then show slightly increased killing activity against *C. albicans* [128]. However, when the LL-25 peptide occurs in the environment, the immunomodulatory properties of LL-37 weaken [128].

The group of AMPs also includes peptides formed as a result of the hydrolysis of larger protein precursors. Such an example may be HK, whose structure contains two sequences, NAT26 and HKH20, that demonstrate AMP features and are susceptible to the action of Saps [129,130]. NAT26 can be inactivated by all Saps except Sap10, while HKH20 is sensitive to Sap9 [131]. Recently, it has been shown that Saps transported by extracellular vesicles are also involved in the cleavage of antibacterial peptides. In this case, a special role is played by Sap5, Sap6, and Sap9, which are involved in the degradation of the NAT26 peptide [41]. Another example of a sequence with AMP properties is the P-113 peptide derived from the previously described His5 peptide [132]. Although its antimicrobial properties were described several years ago, it was only recently demonstrated that, like His5, the P-133 peptide can be cleaved into four shorter fragments with the participation of Saps [133]. Furthermore, the degradation of other truncated and derivative LL-37 peptides with antifungal properties has also been demonstrated by Saps [134]; some of them, including GK-17 peptide characterized in detail, showed less susceptibility to this proteolysis, especially Sap4 and Sap6-dependent [134]. Furthermore, there are also other defense proteins produced by the host that may be degraded by aspartic proteases, including salivary lactoferrin, lactoperoxidase, cathepsin D, α2-macroglobulin, etc. [21,87,135,136].

Nonetheless, the most widely described in the literature is the involvement of Saps in the degradation of proteins belonging to the complement system. In the case of *C. albicans*, it has been proven that Sap1–Sap3 can degrade complement proteins C3b, C4b, and C5, hindering the assembly of the membrane attack complex (MAC) [137] and affecting complement system activation. This is related to the inhibition of *C. albicans* opsonization by C3b and the inability to produce anaphylatoxin C5a [137]. Svoboda et al. described another mechanism of complement avoidance. In this case, Sap2 cleaves factor H (FH), which most likely functions as a bridge between the pathogen and the CR3 receptor on neutrophils [138]. Additionally, Sap2 can also interfere with CR3 and CR4 receptor expression in macrophages [138]. Recently, clinical isolates of *C. albicans* have been analyzed that displayed a new strategy to evade the host immune system [48]. The exchange of the amino acid at position 273 in Sap2 from valine to leucine resulted in escalated proteolytic activity of Sap2273L compared to Sap2273V, suggesting an increased ability of *C. albicans* to evade the complement response and, consequently, an increase in pathogenicity [48]. In the case of *C. parapsilosis*, Sapp1 and Sapp2 cleave complement proteins, including C4b, C3b, and FH [19]. Moreover, for proteinases from *C. tropicalis* (Sapt1-Sapt4) and *C. dubliniensis* (Sapcd1-Sapcd4 and Sapcd7-Sapcd10), the possible involvement in the modulation of the complement system is also concluded on the basis of their similarity to *C. albicans* Saps [18,24]. Recently, the possible proteolytic activity of *C. tropicalis* Sapt1 against C-type lectins, namely, human MBL, CL-11, and DC-SIGN, has been reported. As these molecules are involved in the detection and removal of *Candida* cells, their cleavage by Sapt1 might inhibit the activation of the complement lectin pathway [139].

Importantly, it has also been shown that Saps found in supernatants from *C. albicans* cultures can contribute to the cleavage of human antibodies [136,140,141,142], and different Sap subfamilies may be involved in the cleavage of IgG and IgM. Thus, Sap1–Sap3 are associated with the proteolysis of IgG, and in the case of IgM, the involvement of Sap2 has been observed. Moreover, IgG proteolysis with the participation of Sap2 probably does not lead to the disruption of Fab or F(ab)2 fragments, which allows the maintenance of the functionality of antibodies and also promotes a reduction in *C. albicans* adherence [143]. In the case of IgA, no cleavage has been observed upon contact with Sap2 [143], although proteolysis of this immunoglobulin by other Sap subfamilies is possible [142], similarly as for Sapp1 derived from *C. parapsilosis* [35,47].

### 2.4. Antifungal Resistance

The problems with candidiasis treatments are due to the toxicity of available antifungal drugs, their insufficient effectiveness since only drugs from the polyene group and echinocandins are fungicides, and the development of drug resistance [144]. The search for the relationship between drug sensitivity and the virulence factors of *Candida* species is currently the subject of extensive research. Several reports indicate a positive correlation of *Candida* resistance with Saps production. Kumar and Shukla demonstrated that Sap2 activity was significantly higher in strain resistant to amphotericin B, developed under laboratory conditions, as compared to parent strain *C. albicans* ATCC 10231 [145]. Furthermore, the research by Feng et al. revealed that the expression of gene encoding Sap2 was higher in itraconazole-resistant than in itraconazole-sensitive *C. albicans* strains [146], whereas Kadry et al. reported a strong correlation between Sap9 and Sap10 production and resistance to fluconazole, voriconazole, and 5-fluorocytosine [147]. All tested *C. albicans* isolates resistant to the three above-mentioned drugs and collected from patients with systemic infections produced Sap9, Sap10, or both proteinases [147]. Biofilm formation and the production of Sap2, Sap4, and Sap6 have been shown to be associated with resistance to azoles in patients with vulvovaginal candidiasis [148].

Exposition of *Candida* cells to subinhibitory concentrations of antifungal drugs promotes the development of resistant strains. It has been shown that, under such conditions, the production of Saps may also increase. Wu et al. observed enhanced production of Saps by *C. albicans* isolates grown in a subinhibitory concentration of fluconazole [149]. Cooping et al. found that contact of *C. albicans* with azoles, flucytosine, and caspofungin gave an increase in the gene expression and activity of Sap2 [150]. Barelle et al. proved that genes encoding Sap4–Sap6 proteinases are upregulated in response to the exposition of *C. albicans* to azoles [151].

There are also reports of no or even negative correlation between Saps production and resistance of *Candida* species. El-Houssaini et al. found a negative correlation between minimal inhibitory concentration (MIC) of voriconazole and Saps production by *C. albicans* isolates from vaginal specimens and no correlation between the resistance of these strains to amphotericin B, nystatin, clotrimazole, fluconazole, and micafungin and the activity of Saps [152]. Figueiredo-Carvalho et al. showed no association between the resistance of *C. glabrata* strains to amphotericin B, fluconazole, itraconazole, and micafungin and their proteolytic activity [153]. These studies included 91 strains isolated from different infection sites in two Brazilian hospitals. The existence of a correlation between the production of proteinases and drug resistance was also checked for the highly resistant *Candida* species—*C. auris*—using *C. auris* mutants lacking single genes encoding Sapa1-Sapa7; however, no differences in sensitivity to amphotericin B, fluconazole, or caspofungin were found [26].

### 2.5. Maintenance of Cellular Homeostasis

A strategy that employs *N. glabratus* mutant strains with deletions in one or more YPS-encoding genes has revealed the involvement of yapsins in coping with thermal stress, controlling glucose homeostasis, and maintaining vacuole homeostasis and pH balance [154,155,156,157]. It was shown that the *Ngyps1*Δ mutant cultured at high temperatures had significantly reduced growth, which was reversed upon complementation of the *NgYPS1* gene or after the addition of sorbitol as an osmotic stabilizer [155]. These findings suggest that Yps1 plays a significant role in preserving cell wall integrity and preventing yeast cell lysis under heat stress. Although eight other *NgYPS* genes, namely, *NgYPS2*, *NgYPS4*, and *NgYPS6-11*, were also overexpressed under thermal stress, their role has not been investigated yet [155]. Additional research by Bairwa et al. emphasized a unique function of NgYps1 in yeast survival under conditions of low external pH, as the *Ngyps1*Δ mutant exhibited intracellular acidification, which disrupted normal physiological processes, partly due to the impaired membrane proton pump activity, and also showed an increase in ROS production [157]. Moreover, the role of NgYPS in regulating vacuole pH homeostasis has been revealed, and studies using the NgYPS-deficient mutant strain showed the wide-ranging effects of protease deficiency, including alterations in vacuole size and internal pH, as well as reduced activity of vacuolar membrane V-ATPase and vacuolar carboxypeptidase Y [156]. Finally, recent studies have linked NgYPS with the regulation of the Snf3-dependent low-glucose-sensing pathway, which is crucial in controlling glucose homeostasis in fungal cells [154]. It has been speculated that yeasts create a glucose-depleted environment as a result of the modulation of glucose homeostasis achieved by activating the glycolysis pathway, which consequently leads to the induction of macrophage death [154].

## 3. The Challenges and Opportunities for Developing Novel Strategies to Prevent and Treat *Candida* Infections

### 3.1. Diagnostic Potential of Saps

Invasive candidiasis poses a significant threat to immunocompromised patients and those in intensive care units, and the high rate of fatal cases underscores the urgent need to develop effective, accurate, and fast diagnostic tests. Diagnosing the infection, especially in its initial stages, is difficult because the signs and symptoms of the disease are not always specific or obvious, and currently available tests often lack sensitivity and specificity. The blood culture test widely used for diagnosing microbial infections requires a long time to obtain reliable results, which can cause delays in implementing effective therapeutic treatment [158,159,160].

A particularly interesting group of proteins in terms of their diagnostic potential is currently *Candida* Saps. Since there were some correlations detected between the amount of secreted Saps and the severity of infection [161,162,163], the use of Saps as detection factors during disseminated candidiasis seems rather promising. In addition, it has been shown that their production and secretion are inducible and correlate with invasive disease, thus providing a basis for distinguishing infection from mere colonization [158].

To date, several types of tests have been described that rely on the identification of either Sap antigens or anti-Sap antibodies present in patients’ serum [160,164,165] and urine [158]. The sensitivity of such tests varies depending on the method and the antibodies used; they are relatively simple to perform and do not require advanced equipment. Already several decades ago, Rüchel et al. described the usage of polyclonal antibodies against proteinase in a sandwich-type ELISA to detect proteinase antigen; however, some concerns were raised about the reduced sensitivity due to the formation of Sap–α2-macroglobulin complexes in the circulation [164]. Then, Na and Song compared different types of ELISA tests for detecting Sap antigen in serum, with a marginal advantage in specificity for inhibition/competitive ELISA [165]. In contrast, in the studies by Wang et al., an indirect ELISA method with purified recombinant Sap2 protein as the coating antigen was used [160]. In addition, Morisson et al. showed that the detection of Saps in urine is better compared to the detection in serum in terms of sensitivity [158]. This is most likely due to the capability of the collection of larger volumes of urine than serum, resulting in the ability to concentrate the antigen and overcome the problems associated with the formation of Sap complexes in the circulation. Moreover, studies in a rabbit model revealed that ELISA with urine-based inhibition correlated directly with disease progression, especially in the kidneys [158]. Currently, some novel methods also have been proposed to identify Saps from patients’ samples. Aoki et al. designed a fluorescence-quenched peptide consisting of a fluorophore, quencher, and peptide linker that emits fluorescence when specifically cleaved by Sap, with the sequence highly favoring cleavage by Sap1-3 [166]. Additionally, the indirect ELISA test and Western blot technique were proposed for the detection of *C. albicans* Sap1 recently during serodiagnosis of invasive candidiasis [167]. Furthermore, the diagnostic potential of recombinant Sapp2 from *C. parapsilosis* was also evaluated with immunoblotting and ELISA tests performed using the serum of infected patients [168].

### 3.2. Saps as Components of Anti-Candida Vaccines

Research dedicated to the development of anti-fungal vaccines is progressively advancing, leading to the emergence of potential vaccine candidates and adjuvants. Notably, Sap2 is among the most promising targets.

Initial studies of Sap2-containing vaccines were prompted by reports demonstrating that the vaginal secretions of ovariectomized and estrogen-treated rats, which successfully cleared a primary *C. albicans* infection and demonstrated high resistance to a subsequent yeast challenge, contained anti-mannan or anti-proteinase antibodies [169]. Furthermore, it has been observed that the passive transfer of these secretions to unimmunized rats conferred significant protection against vaginitis [169,170]. A comparable protective effect was detected after the passive post-infection administration of monoclonal anti-Sap and anti-mannan IgM and IgG antibodies, as well as after the active vaginal immunization using a highly purified, polysaccharide-free Sap preparation with complete Freund’s adjuvant [170]. Intriguingly, it was demonstrated that congenitally athymic rats, despite effectively eliminating the primary infection similar to their thymic counterparts, were unable to develop anti-mannan or anti-proteinase antibodies and, hence, were not protected during a subsequent *C. albicans* challenge [170]. Further research conducted by De Bernardis, utilizing estrogen-dependent murine models along with mannoprotein extract and Sap preparation, primarily comprising the Sap2 component, demonstrated that both vaginal and nasal immunization routes effectively stimulate antibody production and significantly enhance protection against subsequent infections [171]. Specifically, in the case of Sap, a notable acceleration in yeast clearance from the vagina was observed within the first week. The combined administration of Sap and the cholera toxin from *Vibrio cholerae*, which has significant potential as mucosal adjuvants, amplified this effect, particularly within the initial five days of infection [171]. Furthermore, it was found that intravaginal immunization of rats with a recombinant, enzymatically inactive Sap2 deprived of 76 amino acids at the N-terminus led to the local production of monoclonal anti-Sap2 IgG and IgA antibodies, thereby providing the animals with protection against yeast infection [172].

To develop a highly immunogenic and clinically acceptable vaccine for the prevention of recurrent vulvovaginal infections, a truncated recombinant *C. albicans* Sap2 (with amino acids 77–400) was utilized in combination with virosome technology. The resulting construct was named PEV7 [173]. Virosomes, which are structures recreated in vitro from the influenza virus, lack viral RNA but maintain the virus’s ability to bind to and penetrate target cells, including the unique pH-dependent fusion activity mediated by hemagglutinin. The employment of virosomes as carriers and adjuvants significantly enhances antigen presentation [174]. In the initial phase of the study, it was demonstrated that immunization with PEV7 triggers a significantly stronger anti-Sap2 IgG antibody response in rat serum compared to immunization with an equivalent amount of recombinant Sap2 not bound to virosomes [172]. Although it was shown that the levels of anti-Sap2 antibodies, both IgG and IgA, in the vaginal fluid of rats immunized intravaginally with PEV7 were significantly lower than those observed after intramuscular immunization, in both cases, the levels were significantly higher than those resulting from the administration of the uncovered recombinant protein. This observation underscored the pivotal role of the virosome in eliciting a robust immune response [172]. Furthermore, it has been demonstrated that rats immunized intravaginally with the PEV7 vaccine exhibited significant protection against yeasts, with the infection being cleared at least seven days sooner in this group of animals than in the control group [173]. Notably, in the toxicological study conducted, the repeated administration of the PEV7 preparation to rats did not cause any clinical outcomes, both functional and behavioral [173]. In the first phase of clinical trials (registration number NCT01067131), PEV7 was tested as a vaccine for recurrent vulvovaginal candidiasis (RVVC). The first group tested comprised women of reproductive age, and the vaccines were delivered via two distinct methods, either as intramuscular injections or vaginal tablets. The primary endpoint of the study revealed that all vaccinated women rapidly developed a specific memory of B lymphocytes. Subsequently, the secondary endpoint, recorded six months after the vaccination cycle, indicated a sustained high antibody titer, thereby affirming the considerable potential of PEV7 as a therapeutic vaccine [175].

Using an immunoinformatic approach, eleven epitopes for B and T cells were pinpointed on the surface of Sapt2 [176] and eight epitopes on the surface of the CdSap2 [177]. These epitopes, being highly antigenic, non-allergenic, and non-toxic, and in the case of *C. dubliniensis*, demonstrating the potential to induce IL-2, IL-4, and IFN-γ, appear to be promising candidates for vaccines. Through molecular docking and molecular dynamics simulations, it was established that the proposed vaccine constructs can bind stably to toll-like receptor 5 (TLR5), and, in the case of Sapt2, also to the major histocompatibility complex (MHC-I) [176,177]. Furthermore, in vitro studies confirmed the role of recombinant Sapp2 (amino acids 77-398) and Sapt2 (amino acids 90-397), used in conjunction with alum as an adjuvant [178]. The greatest benefits in terms of mouse survival were noticed following immunization with the Sapp2 preparation. These mice exhibited elevated titers of IgM and IgG antibodies and increased levels of Th1, Th2, and Th17 cytokines, underscoring the immunomodulatory attributes of Sapp2. Additionally, the passive transfer of serum from Sapp2-immunized mice markedly enhanced the survival of naive mice [178]. Histopathological analysis revealed an increase in neutrophil recruitment in mice immunized with Sapp2 and Sapt2 and an enhancement in neutrophil-mediated killing in the presence of serum from immunized mice (at 73% and 64% for Sapp2 and Sapt2, respectively) compared to sham-immunized serum, indicating the increased opsonic activity of antibodies produced by proteinases. Interestingly, serum from Sapp2-immunized mice has been shown to inhibit biofilm formation by *C. tropicalis* [178].

### 3.3. Protease Inhibitors as Prospective Agents Accompanying the Treatment of Candidiasis

Since inhibitors of proteinases present in human plasma, other fluids, or epidermal tissues are hydrolyzed by Saps during fungal infection [86,95,96,128], in advanced stages of candidiasis, their activity is not sufficient, and additional molecules that impact Saps action are required to control infection. Therefore, the potential of other compounds, synthetic or natural, often of external origin, has been investigated to inhibit the enzymatic activity of Saps. Numerous naturally present molecules show an inhibitory effect on proteases released by microorganisms during infections. One example is an antimicrobial enzyme present in human saliva, lysozyme, which, when used in lower concentrations, has an impact on the reduction in Sap2 production by *C. albicans* [179]. Additionally, the isolated peptide fragment of glyceraldehyde 3-phosphate dehydrogenase (GAPDH) from human placental tissue was reported to inhibit Sap1 and Sap2 activity, but no effect was observed for Sap3 [180]. Among peptide inhibitors of aspartic proteinases, pepstatin A was described over 30 years ago [181]. The usage of this inhibitor resulted in the inhibition of Sap activity, reduced virulence of fungal cells, and impaired adhesion to host cells; however, this molecule was rapidly metabolized and inhibited the activity of other enzymes, which impeded its application as a drug in the nonmodified form [181]. Nonetheless, the structure of pepstatin A was a promising basis for designing and synthesizing other molecules with high efficiency in the inhibition of Sap1, Sap2, Sap3, Sap5, and Sap6 [182,183]. The analysis of the complexes formed by protease and inhibitor provides crucial information on the differences among Saps produced by various *Candida* species, including Sapp1, Sapt1, and Sap2, that explain possible differences in substrate specificities [184]. Some potential proteinase inhibitors based on the structure of pepstatin A were shown to have low affinities for Sapp1, thus affecting their effectiveness [185].

Additionally, some medicines being approved for the treatment of other diseases were examined for their effect on fungi. Research on drugs used in therapies in HIV-infected patients revealed their potential use in the treatment of candidiasis. Substances acting as HIV protease inhibitors, such as ritonavir, indinavir, saquinavir, nelfinavir, amprenavir, or lopinavir, were reported to have an inhibitory effect on *C. albicans* Sap activity in in vitro and in vivo tests [186,187,188,189,190,191,192,193,194] and some of them also on the activity of Saps produced by *C. tropicalis*, *C. parapsilosis*, and *C. lusitaniae* [17,195]. These drugs demonstrated different inhibitory potentials depending on the concentrations used and the conditions applied. Among them, ritonavir showed the strongest effect [190,191], while saquinavir revealed not only inhibition of Sap activity but also fungicidal potential [186], and indinavir was specific to Sap2 [190,191], similar to lopinavir [194]. The activity of Sap4, Sap5, and Sap6 was not affected by the above-mentioned inhibitors [190,191]; likewise, pepstatin A did not fully inhibit Sap7 [110] or Sap9 and Sap10 [46]. The inhibition of *C. parapsilosis* Saps activity was observed for ritonavir but not for saquinavir [195]. These observations clearly prove that inhibition of Saps has to be directed and related to their specificity and conditions optimal for enzymatic activity. 

In further studies, the potential use of different inhibitory molecules derived from plants [196,197,198] or microorganisms [199] was considered. For some compounds from an ethanol extract of *Lycopodium cernuum*, an inhibitory effect against Saps was observed, while others did not present such properties [196]. The combination of fluoxetine and azole was indicated as an inhibitor of proteolytic activity due to the potential effect on the downregulation of genes encoding Saps [200]. Metabolites of Basidiomycete, such as laccaridiones B or aureoquinone, inhibited the production of Saps or reduced their release [201]. A similar mechanism of action was presented for one of the flavonoids—phloretin. In the presence of phloretin, Sap1 and Sap2 secretion was reduced due to attenuation of gene expression [198].

Also, the usage of artificial materials as inhibitors of Saps activity was considered. Triangular gold nanoparticles have been reported to inhibit Sap2 activity in a dose- and time-dependent manner. Further research indicated better functional properties of nanoparticles conjugated with synthesized peptide ligands for Sap2 [202]. Additionally, the compounds from a group of renin inhibitors, including A-70450 or A-79912, also inhibited the enzymatic activity of Sap2, but in vivo experiments revealed no protective effect [203,204,205,206]. Another renin inhibitor and drug used for hypertension treatment, aliskiren, also significantly reduced Saps activity, especially Sap2 [207]. In addition, in recent studies, structural analysis of compounds from *Piper crocatum* extract and molecular modeling revealed two potential ligands for Sap5, CHEMBL216163 and MLS000557666, forming bonds with enzymes that are considered important for efficient ligand binding [208].

Despite the preliminary promising results, the considered use of proteinase inhibitors in the treatment of candidiasis needs further detailed analysis of the structures, interaction mechanisms, and functional effects of proposed molecules.

## 4. Recommendations and Suggestions for Future Research Directions and Priorities on the Role of Saps in *Candida* Infections

**Figure 4:**
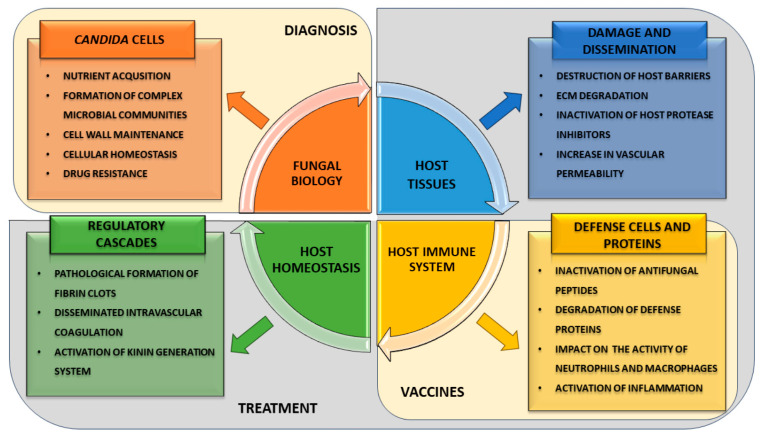
Graphic summary of the functions of *Candida* spp. aspartyl proteases and the key challenges in current research, which include the search for diagnostic biomarkers and alternative treatments.

The functionalities of Sap proteases are crucial for many aspects of the biology and pathophysiology of *Candida* fungi, thus connecting with the potential to use their presence and activity for the prevention, diagnosis, and treatment of fungal infections (Figure 4). To increase the understanding of the multifaceted role that Saps play in *Candida* infections and to propose more effective diagnostic, preventive, and therapeutic strategies against candidiases, the multidisciplinary approaches bringing together microbiology, immunology, biochemistry, genetics, and clinical research are likely to result with the most comprehensive insights. In the design of effective antifungal therapies, the investigation of the potential of Saps as targets for small molecule inhibitors and as vaccines’ components is particularly important. Additionally, the exploration of the efficacy of Saps as biomarkers for the diagnosis and prognosis of *Candida* infections and for monitoring therapeutic responses is one of the current challenges. Therefore, also unveiling the molecular mechanisms by which Saps contribute to *Candida* pathogenicity, including their role in host tissue invasion and immune evasion, is essential.

Currently, most of the published detailed data relate to Saps produced by *C. albicans* and *C. parapsilosis*. There are still several issues that are insufficiently well understood for proteinases of other *Candida* species that currently pose a clinical threat, including *C. auris*. One of these is the characterization of the specific functions and regulatory mechanisms of Saps during various stages of fungal infection and in different host environments, including the genetic and epigenetic factors that regulate *SAP* gene expression and Sap protein activity in response to environmental cues and host signals. Investigation of the interactions between Saps and host cell receptors, extracellular matrix components, and immune cells might help us understand how these relations facilitate infection and inflammation.

The application of systems biology approaches to integrate data from genomic, proteomic, and metabolomic studies may provide new insight into their importance and a better understanding of the role of Saps in *Candida* biology and pathogenesis. Additionally, consideration of the results from clinical studies to evaluate the clinical relevance of Saps in different types of *Candida* infections and in diverse patient populations, as well as using computational modeling to predict the impact of Saps on the dynamics of *Candida* infections and to simulate the effects of potential therapeutic interventions, may open new perspectives in the approach for combating fungal infections.

## References

[1] Chen C., Huang X., Sun J., Dudeja P.K. (2018). Candida albicans Commensalism and Human Diseases. Mechanisms Underlying Host-Microbiome Interactions in Pathophysiology of Human Diseases.

[2] Kumamoto C.A., Gresnigt M.S., Hube B. (2020). The Gut, the Bad and the Harmless: *Candida albicans* as a Commensal and Opportunistic Pathogen in the Intestine. Curr. Opin. Microbiol..

[3] Lopes J.P., Lionakis M.S. (2022). Pathogenesis and Virulence of *Candida albicans*. Virulence.

[4] Proctor D.M., Drummond R.A., Lionakis M.S., Segre J.A. (2023). One Population, Multiple Lifestyles: Commensalism and Pathogenesis in the Human Mycobiome. Cell Host Microbe.

[5] Logan C., Martin-Loeches I., Bicanic T. (2020). Invasive Candidiasis in Critical Care: Challenges and Future Directions. Intensive Care Med..

[6] McCarty T.P., White C.M., Pappas P.G. (2021). Candidemia and Invasive Candidiasis. Infect. Dis. Clin. N. Am..

[7] Soriano A., Honore P.M., Puerta-Alcalde P., Garcia-Vidal C., Pagotto A., Gonçalves-Bradley D.C., Verweij P.E. (2023). Invasive Candidiasis: Current Clinical Challenges and Unmet Needs in Adult Populations. J. Antimicrob. Chemother..

[8] Bongomin F., Gago S., Oladele R., Denning D. (2017). Global and Multi-National Prevalence of Fungal Diseases—Estimate Precision. J. Fungi.

[9] Pfaller M.A., Diekema D.J. (2007). Epidemiology of Invasive Candidiasis: A Persistent Public Health Problem. Clin. Microbiol. Rev..

[10] Arendrup M.C. (2013). *Candida* and Candidaemia. Susceptibility and Epidemiology. Dan. Med. J..

[11] Quindós G., Marcos-Arias C., San-Millán R., Mateo E., Eraso E. (2018). The Continuous Changes in the Aetiology and Epidemiology of Invasive Candidiasis: From Familiar *Candida albicans* to Multiresistant *Candida auris*. Int. Microbiol..

[12] Sharma M., Chakrabarti A. (2023). Candidiasis and Other Emerging Yeasts. Curr. Fungal Infect. Rep..

[13] Polke M., Hube B., Jacobsen I.D. (2015). *Candida* Survival Strategies. Adv. Appl. Microbiol..

[14] Loaiza-Loeza S., Parra-Ortega B., Cancino-Díaz J.C., Illades-Aguiar B., Hernández-Rodríguez C.H., Villa-Tanaca L. (2009). Differential Expression of *Candida dubliniensis*-Secreted Aspartyl Proteinase Genes (*CdSAP1–4*) under Different Physiological Conditions and during Infection of a Keratinocyte Culture. FEMS Immunol. Med. Microbiol..

[15] Naglik J.R., Challacombe S.J., Hube B. (2003). *Candida albicans* Secreted Aspartyl Proteinases in Virulence and Pathogenesis. Microbiol. Mol. Biol. Rev..

[16] Monod M., Togni G., Hube B., Sanglard D. (1994). Multiplicity of Genes Encoding Secreted Aspartic Proteinases in *Candida* Species. Mol. Microbiol..

[17] Pichová I., Pavlícková L., Dostál J., Dolejsí E., Hrusková-Heidingsfeldová O., Weber J., Ruml T., Soucek M. (2001). Secreted Aspartic Proteases of *Candida albicans*, *Candida tropicalis*, *Candida parapsilosis* and *Candida lusitaniae*. Inhibition with Peptidomimetic Inhibitors. Eur. J. Biochem..

[18] Parra-Ortega B., Cruz-Torres H., Villa-Tanaca L., Hernández-Rodríguez C. (2009). Phylogeny and Evolution of the Aspartyl Protease Family from Clinically Relevant *Candida* Species. Mem. Inst. Oswaldo Cruz.

[19] Singh D.K., Németh T., Papp A., Tóth R., Lukácsi S., Heidingsfeld O., Dostal J., Vágvölgyi C., Bajtay Z., Józsi M. (2019). Functional Characterization of Secreted Aspartyl Proteases in *Candida parapsilosis*. mSphere.

[20] Kwon-Chung K.J., Lehman D., Good C., Magee P.T. (1985). Genetic Evidence for Role of Extracellular Proteinase in Virulence of *Candida albicans*. Infect. Immun..

[21] Hube B. (1996). *Candida albicans* Secreted Aspartyl Proteinases. Curr. Top. Med. Mycol..

[22] Fusek M., Smith E.A., Monod M., Foundling S.I. (1993). *Candida parapsilosis* Expresses and Secretes Two Aspartic Proteinases. FEBS Lett..

[23] Silva S., Henriques M., Oliveira R., Azeredo J., Malic S., Hooper S.J., Williams D.W. (2009). Characterization of *Candida parapsilosis* Infection of an *in Vitro* Reconstituted Human Oral Epithelium. Eur. J. Oral Sci..

[24] Zaugg C., Borg-von Zepelin M., Reichard U., Sanglard D., Monod M. (2001). Secreted Aspartic Proteinase Family of *Candida tropicalis*. Infect. Immun..

[25] Moran G. (2004). Comparative Genomics Using *Candida albicans* DNA Microarrays Reveals Absence and Divergence of Virulence-Associated Genes in *Candida dubliniensis*. Microbiology.

[26] Kim J.-S., Lee K.-T., Bahn Y.-S. (2023). Secreted Aspartyl Protease 3 Regulated by the Ras/cAMP/PKA Pathway Promotes the Virulence of *Candida auris*. Front. Cell. Infect. Microbiol..

[27] Hube B., Sanglard D., Odds F.C., Hess D., Monod M., Schäfer W., Brown A.J., Gow N.A. (1997). Disruption of Each of the Secreted Aspartyl Proteinase Genes *SAP1*, *SAP2*, and *SAP3* of *Candida albicans* Attenuates Virulence. Infect. Immun..

[28] Staib P., Lermann U., Blaβ-Warmuth J., Degel B., Würzner R., Monod M., Schirmeister T., Morschhäuser J. (2008). Tetracycline-Inducible Expression of Individual Secreted Aspartic Proteases in *Candida albicans* Allows Isoenzyme-Specific Inhibitor Screening. Antimicrob. Agents Chemother..

[29] Gil-Bona A., Monteoliva L., Gil C. (2015). Global Proteomic Profiling of the Secretome of *Candida albicans* Ecm33 Cell Wall Mutant Reveals the Involvement of Ecm33 in Sap2 Secretion. J. Proteome Res..

[30] Kaur R., Ma B., Cormack B.P. (2007). A Family of Glycosylphosphatidylinositol-Linked Aspartyl Proteases Is Required for Virulence of *Candida glabrata*. Proc. Natl. Acad. Sci. USA.

[31] Kidd S.E., Abdolrasouli A., Hagen F. (2023). Fungal Nomenclature: Managing Change Is the Name of the Game. Open Forum Infect. Dis..

[32] Newport G., Agabian N. (1997). KEX2 Influences *Candida albicans* Proteinase Secretion and Hyphal Formation. J. Biol. Chem..

[33] Togni G., Sanglard D., Quadroni M., Foundling S.I., Monod M. (1996). Acid Proteinase Secreted by *Candida tropicalis*: Functional Analysis of Preproregion Cleavages in *C. tropicalis* and Saccharomyces Cerevisiae. Microbiology.

[34] Dostál J., Dlouhá H., Maloň P., Pichová I., Hrušková-Heidingsfeldová O. (2005). The Precursor of Secreted Aspartic Proteinase Sapp1p from *Candida parapsilosis* Can Be Activated Both Autocatalytically and by a Membrane-Bound Processing Proteinase. Biol. Chem..

[35] Merkerová M., Dostál J., Hradilek M., Pichová I., Hrusková-Heidingsfeldová O. (2006). Cloning and Characterization of Sapp2p, the Second Aspartic Proteinase Isoenzyme from *Candida parapsilosis*. FEMS Yeast Res..

[36] Koelsch G., Tang J., Loy J.A., Monod M., Jackson K., Foundling S.I., Lin X. (2000). Enzymic Characteristics of Secreted Aspartic Proteases of *Candida albicans*. Biochim. Biophys. Acta (BBA)-Protein Struct. Mol. Enzymol..

[37] Albrecht A., Felk A., Pichova I., Naglik J.R., Schaller M., de Groot P., Maccallum D., Odds F.C., Schäfer W., Klis F. (2006). Glycosylphosphatidylinositol-Anchored Proteases of *Candida albicans* Target Proteins Necessary for Both Cellular Processes and Host-Pathogen Interactions. J. Biol. Chem..

[38] Zarnowski R., Sanchez H., Covelli A.S., Dominguez E., Jaromin A., Bernhardt J., Mitchell K.F., Heiss C., Azadi P., Mitchell A. (2018). *Candida albicans* Biofilm–Induced Vesicles Confer Drug Resistance through Matrix Biogenesis. PLoS Biol..

[39] Kulig K., Karnas E., Woznicka O., Kuleta P., Zuba-Surma E., Pyza E., Osyczka A., Kozik A., Rapala-Kozik M., Karkowska-Kuleta J. (2022). Insight into the Properties and Immunoregulatory Effect of Extracellular Vesicles Produced by *Candida glabrata*, *Candida parapsilosis*, and *Candida tropicalis* Biofilms. Front. Cell. Infect. Microbiol..

[40] Karkowska-Kuleta J., Kulig K., Karnas E., Zuba-Surma E., Woznicka O., Pyza E., Kuleta P., Osyczka A., Rapala-Kozik M., Kozik A. (2020). Characteristics of Extracellular Vesicles Released by the Pathogenic Yeast-Like Fungi *Candida glabrata*, *Candida parapsilosis* and *Candida tropicalis*. Cells.

[41] Karkowska-Kuleta J., Kulig K., Bras G., Stelmaszczyk K., Surowiec M., Kozik A., Karnas E., Barczyk-Woznicka O., Zuba-Surma E., Pyza E. (2023). *Candida albicans* Biofilm-Derived Extracellular Vesicles Are Involved in the Tolerance to Caspofungin, Biofilm Detachment, and Fungal Proteolytic Activity. J. Fungi.

[42] Martínez-López R., Hernáez M.L., Redondo E., Calvo G., Radau S., Pardo M., Gil C., Monteoliva L. (2022). *Candida albicans* Hyphal Extracellular Vesicles Are Different from Yeast Ones, Carrying an Active Proteasome Complex and Showing a Different Role in Host Immune Response. Microbiol. Spectr..

[43] Fusek M., Smith E.A., Monod M., Dunn B.M., Foundling S.I. (1994). Extracellular Aspartic Proteinases from *Candida albicans*, *Candida tropicalis*, and *Candida parapsilosis* Yeasts Differ Substantially in Their Specificities. Biochemistry.

[44] Aoki W., Kitahara N., Miura N., Morisaka H., Yamamoto Y., Kuroda K., Ueda M. (2011). Comprehensive Characterization of Secreted Aspartic Proteases Encoded by a Virulence Gene Family in *Candida albicans*. J. Biochem..

[45] Gogol M., Bochenska O., Zawrotniak M., Karkowska-Kuleta J., Zajac D., Rapala-Kozik M., Chakraborti S., Dhalla N.S. (2017). Roles of *Candida albicans* Aspartic Proteases in Host-Pathogen Interactions. Pathophysiological Aspects of Proteases.

[46] Schild L., Heyken A., de Groot P.W.J., Hiller E., Mock M., de Koster C., Horn U., Rupp S., Hube B. (2011). Proteolytic Cleavage of Covalently Linked Cell Wall Proteins by *Candida albicans* Sap9 and Sap10. Eukaryot. Cell.

[47] Hrušková-Heidingsfeldová O., Dostál J., Majer F., Havlíková J., Hradilek M., Pichová I. (2009). Two Aspartic Proteinases Secreted by the Pathogenic Yeast *Candida Parapsilosis* Differ in Expression Pattern and Catalytic Properties. Biol. Chem..

[48] Lin L., Wang M., Zeng J., Mao Y., Qin R., Deng J., Ouyang X., Hou X., Sun C., Wang Y. (2023). Sequence Variation of *Candida albicans* Sap2 Enhances Fungal Pathogenicity via Complement Evasion and Macrophage M2-Like Phenotype Induction. Adv. Sci..

[49] Naglik J., Albrecht A., Bader O., Hube B. (2004). *Candida albicans* Proteinases and Host/Pathogen Interactions. Cell. Microbiol..

[50] Van Eck N.J., Waltman L. (2011). Text Mining and Visualization Using VOSviewer. arXiv.

[51] Ponde N.O., Lortal L., Ramage G., Naglik J.R., Richardson J.P. (2021). *Candida albicans* Biofilms and Polymicrobial Interactions. Crit. Rev. Microbiol..

[52] Pereira R., Santos Fontenelle R.O., Brito E.H.S., Morais S.M. (2021). Biofilm of *Candida albicans*: Formation, Regulation and Resistance. J. Appl. Microbiol..

[53] Winter M.B., Salcedo E.C., Lohse M.B., Hartooni N., Gulati M., Sanchez H., Takagi J., Hube B., Andes D.R., Johnson A.D. (2016). Global Identification of Biofilm-Specific Proteolysis in *Candida albicans*. mBio.

[54] Lohse M.B., Gulati M., Johnson A.D., Nobile C.J. (2018). Development and Regulation of Single- and Multi-Species *Candida albicans* Biofilms. Nat. Rev. Microbiol..

[55] Nobile C.J., Fox E.P., Nett J.E., Sorrells T.R., Mitrovich Q.M., Hernday A.D., Tuch B.B., Andes D.R., Johnson A.D. (2012). A Recently Evolved Transcriptional Network Controls Biofilm Development in *Candida albicans*. Cell.

[56] Nailis H., Kucharíková S., Ricicová M., Van Dijck P., Deforce D., Nelis H., Coenye T. (2010). Real-Time PCR Expression Profiling of Genes Encoding Potential Virulence Factors in *Candida albicans* Biofilms: Identification of Model-Dependent and -Independent Gene Expression. BMC Microbiol..

[57] Joo M.Y., Shin J.H., Jang H.-C., Song E.S., Kee S.J., Shin M.G., Suh S.P., Ryang D.W. (2013). Expression of *SAP5* and *SAP9* in *Candida albicans* Biofilms: Comparison of Bloodstream Isolates with Isolates from Other Sources. Med. Mycol..

[58] Tumbarello M., Fiori B., Trecarichi E.M., Posteraro P., Losito A.R., De Luca A., Sanguinetti M., Fadda G., Cauda R., Posteraro B. (2012). Risk Factors and Outcomes of Candidemia Caused by Biofilm-Forming Isolates in a Tertiary Care Hospital. PLoS ONE.

[59] Rajendran R., May A., Sherry L., Kean R., Williams C., Jones B.L., Burgess K.V., Heringa J., Abeln S., Brandt B.W. (2016). Integrating *Candida albicans* Metabolism with Biofilm Heterogeneity by Transcriptome Mapping. Sci. Rep..

[60] Wu H., Downs D., Ghosh K., Ghosh A.K., Staib P., Monod M., Tang J. (2013). *Candida albicans* Secreted Aspartic Proteases 4-6 Induce Apoptosis of Epithelial Cells by a Novel Trojan Horse Mechanism. FASEB J..

[61] Kumar R., Breindel C., Saraswat D., Cullen P.J., Edgerton M. (2017). *Candida albicans* Sap6 Amyloid Regions Function in Cellular Aggregation and Zinc Binding, and Contribute to Zinc Acquisition. Sci. Rep..

[62] Fox E.P., Bui C.K., Nett J.E., Hartooni N., Mui M.C., Andes D.R., Nobile C.J., Johnson A.D. (2015). An Expanded Regulatory Network Temporally Controls *Candida albicans* Biofilm Formation. Mol. Microbiol..

[63] Li F., Svarovsky M.J., Karlsson A.J., Wagner J.P., Marchillo K., Oshel P., Andes D., Palecek S.P. (2007). Eap1p, an Adhesin That Mediates *Candida albicans* Biofilm Formation In Vitro and In Vivo. Eukaryot. Cell.

[64] Nobbs A.H., Margaret Vickerman M., Jenkinson H.F. (2010). Heterologous Expression of *Candida albicans* Cell Wall-Associated Adhesins in *Saccharomyces cerevisiae* Reveals Differential Specificities in Adherence and Biofilm Formation and in Binding Oral *Streptococcus gordonii*. Eukaryot. Cell.

[65] Fox S.J., Shelton B.T., Kruppa M.D. (2013). Characterization of Genetic Determinants That Modulate *Candida albicans* Filamentation in the Presence of Bacteria. PLoS ONE.

[66] Ramsook C.B., Tan C., Garcia M.C., Fung R., Soybelman G., Henry R., Litewka A., O’Meally S., Otoo H.N., Khalaf R.A. (2010). Yeast Cell Adhesion Molecules Have Functional Amyloid-Forming Sequences. Eukaryot. Cell.

[67] Dutton L.C., Jenkinson H.F., Lamont R.J., Nobbs A.H. (2016). Role of *Candida albicans* Secreted Aspartyl Protease Sap9 in Interkingdom Biofilm Formation. Pathog. Dis..

[68] Silverman R.J., Nobbs A.H., Vickerman M.M., Barbour M.E., Jenkinson H.F. (2010). Interaction of *Candida albicans* Cell Wall Als3 Protein with *Streptococcus gordonii* SspB Adhesin Promotes Development of Mixed-Species Communities. Infect. Immun..

[69] Nobbs A.H., Jenkinson H.F. (2015). Interkingdom Networking within the Oral Microbiome. Microbes Infect..

[70] Uppuluri P., Chaturvedi A.K., Srinivasan A., Banerjee M., Ramasubramaniam A.K., Köhler J.R., Kadosh D., Lopez-Ribot J.L. (2010). Dispersion as an Important Step in the *Candida albicans* Biofilm Developmental Cycle. PLoS Pathog..

[71] Uppuluri P., Pierce C.G., Thomas D.P., Bubeck S.S., Saville S.P., Lopez-Ribot J.L. (2010). The Transcriptional Regulator Nrg1p Controls *Candida albicans* Biofilm Formation and Dispersion. Eukaryot Cell.

[72] Uppuluri P., Acosta Zaldívar M., Anderson M.Z., Dunn M.J., Berman J., Lopez Ribot J.L., Köhler J.R. (2018). *Candida albicans* Dispersed Cells Are Developmentally Distinct from Biofilm and Planktonic Cells. mBio.

[73] Munro C.A., Hube B. (2002). Anti-Fungal Therapy at the HAART of Viral Therapy. Trends Microbiol..

[74] Cassone A., Cauda R. (2002). HIV Proteinase Inhibitors: Do They Really Work against *Candida* in a Clinical Setting?. Trends Microbiol..

[75] Jalal M., Ansari M.A., Alzohairy M.A., Ali S.G., Khan H.M., Almatroudi A., Siddiqui M.I. (2019). Anticandidal Activity of Biosynthesized Silver Nanoparticles: Effect on Growth, Cell Morphology, and Key Virulence Attributes of *Candida* Species. Int. J. Nanomed..

[76] Lohse M.B., Gulati M., Craik C.S., Johnson A.D., Nobile C.J. (2020). Combination of Antifungal Drugs and Protease Inhibitors Prevent *Candida albicans* Biofilm Formation and Disrupt Mature Biofilms. Front. Microbiol..

[77] Colina A.R., Aumont F., Deslauriers N., Belhumeur P., De Repentigny L. (1996). Evidence for Degradation of Gastrointestinal Mucin by *Candida albicans* Secretory Aspartyl Proteinase. Infect. Immun..

[78] De Repentigny L., Aumont F., Bernard K., Belhumeur P. (2000). Characterization of Binding of *Candida albicans* to Small Intestinal Mucin and Its Role in Adherence to Mucosal Epithelial Cells. Infect. Immun..

[79] Villar C.C., Kashleva H., Nobile C.J., Mitchell A.P., Dongari-Bagtzoglou A. (2007). Mucosal Tissue Invasion by *Candida albicans* Is Associated with E-Cadherin Degradation, Mediated by Transcription Factor Rim101p and Protease Sap5p. Infect. Immun..

[80] Tomlin H., Piccinini A.M. (2018). A Complex Interplay between the Extracellular Matrix and the Innate Immune Response to Microbial Pathogens. Immunology.

[81] Naglik J.R., Rodgers C.A., Shirlaw P.J., Dobbie J.L., Fernandes-Naglik L.L., Greenspan D., Agabian N., Challacombe S.J. (2003). Differential Expression of *Candida albicans* Secreted Aspartyl Proteinase and Phospholipase B Genes in Humans Correlates with Active Oral and Vaginal Infections. J. Infect. Dis..

[82] Ray T.L., Payne C.D. (1990). Comparative Production and Rapid Purification of *Candida* Acid Proteinase from Protein-Supplemented Cultures. Infect. Immun..

[83] Morschhäuser J., Virkola R., Korhonen T.K., Hacker J. (2006). Degradation of Human Subendothelial Extracellular Matrix by Proteinase-Secreting *Candida albicans*. FEMS Microbiol. Lett..

[84] Zhang W., Liu Y., Zhang H. (2021). Extracellular Matrix: An Important Regulator of Cell Functions and Skeletal Muscle Development. Cell Biosci..

[85] Kaminishi H., Hamatake H., Cho T., Tamaki T., Suenaga N., Fujii T., Hagihara Y., Maeda H. (1994). Activation of Blood Clotting Factors by Microbial Proteinases. FEMS Microbiol. Lett..

[86] Rüchel R. (1983). On the Renin-like Activity of Candida Proteinases and Activation of Blood Coagulation in vitro. Zentralbl Bakteriol. Mikrobiol. Hyg. A Med. Mikrobiol. Infekt. Parasitol..

[87] Kaminishi H., Miyaguchi H., Tamaki T., Suenaga N., Hisamatsu M., Mihashi I., Matsumoto H., Maeda H., Hagihara Y. (1995). Degradation of Humoral Host Defense by *Candida albicans* Proteinase. Infect. Immun..

[88] Schmaier A.H. (2016). The Contact Activation and Kallikrein/Kinin Systems: Pathophysiologic and Physiologic Activities. J. Thromb. Haemost..

[89] Frick I.-M., Björck L., Herwald H. (2007). The Dual Role of the Contact System in Bacterial Infectious Disease. Thromb. Haemost..

[90] Kaminishi H., Tanaka M., Cho T., Maeda H., Hagihara Y. (1990). Activation of the Plasma Kallikrein-Kinin System by *Candida albicans* Proteinase. Infect. Immun..

[91] Rapala-Kozik M., Karkowska-Kuleta J., Ryzanowska A., Golda A., Barbasz A., Faussner A., Kozik A. (2010). Degradation of Human Kininogens with the Release of Kinin Peptides by Extracellular Proteinases of *Candida* spp.. Biol. Chem..

[92] Bras G., Bochenska O., Rapala-Kozik M., Guevara-Lora I., Faussner A., Kozik A. (2012). Extracellular Aspartic Protease SAP2 of *Candida albicans* Yeast Cleaves Human Kininogens and Releases Proinflammatory Peptides, Met-Lys-Bradykinin and Des-Arg(9)-Met-Lys-Bradykinin. Biol. Chem..

[93] Bras G., Bochenska O., Rapala-Kozik M., Guevara-Lora I., Faussner A., Kamysz W., Kozik A. (2013). Release of Biologically Active Kinin Peptides, Met-Lys-Bradykinin and Leu-Met-Lys-Bradykinin from Human Kininogens by Two Major Secreted Aspartic Proteases of *Candida parapsilosis*. Peptides.

[94] Kozik A., Gogol M., Bochenska O., Karkowska-Kuleta J., Wolak N., Kamysz W., Aoki W., Ueda M., Faussner A., Rapala-Kozik M. (2015). Kinin Release from Human Kininogen by 10 Aspartic Proteases Produced by Pathogenic Yeast *Candida albicans*. BMC Microbiol..

[95] Gogol M., Ostrowska D., Klaga K., Bochenska O., Wolak N., Aoki W., Ueda M., Kozik A., Rapala-Kozik M. (2016). Inactivation of A1-Proteinase Inhibitor by *Candida albicans* Aspartic Proteases Favors the Epithelial and Endothelial Cell Colonization in the Presence of Neutrophil Extracellular Traps. Acta Biochim. Pol..

[96] Tsushima H., Mine H., Kawakami Y., Hyodoh F., Ueki A. (1994). *Candida albicans* Aspartic Proteinase Cleaves and Inactivates Human Epidermal Cysteine Proteinase Inhibitor, Cystatin A. Microbiology.

[97] Müller-Esterl W. (1987). Novel Functions of the Kininogens. Semin. Thromb. Hemost..

[98] Lecaille F., Brömme D., Lalmanach G. (2008). Biochemical Properties and Regulation of Cathepsin K Activity. Biochimie.

[99] Bradford H.N., Jameson B.A., Adam A.A., Wassell R.P., Colman R.W. (1993). Contiguous Binding and Inhibitory Sites on Kininogens Required for the Inhibition of Platelet Calpain. J. Biol. Chem..

[100] Borregaard N. (2010). Neutrophils, from Marrow to Microbes. Immunity.

[101] Appelberg R. (2007). Neutrophils and Intracellular Pathogens: Beyond Phagocytosis and Killing. Trends Microbiol..

[102] Kannan S. (2002). Role of Protease-Activated Receptors in Neutrophil Degranulation. Med. Hypotheses.

[103] Araźna M., Pruchniak M.P., Demkow U. (2015). Reactive Oxygen Species, Granulocytes, and NETosis. Adv. Exp. Med. Biol..

[104] Branzk N., Lubojemska A., Hardison S.E., Wang Q., Gutierrez M.G., Brown G.D., Papayannopoulos V. (2014). Neutrophils Sense Microbe Size and Selectively Release Neutrophil Extracellular Traps in Response to Large Pathogens. Nat. Immunol..

[105] Zawrotniak M., Wojtalik K., Rapala-Kozik M. (2019). Farnesol, a Quorum-Sensing Molecule of *Candida albicans* Triggers the Release of Neutrophil Extracellular Traps. Cells.

[106] Zawrotniak M., Bochenska O., Karkowska-Kuleta J., Seweryn-Ozog K., Aoki W., Ueda M., Kozik A., Rapala-Kozik M. (2017). Aspartic Proteases and Major Cell Wall Components in *Candida albicans* Trigger the Release of Neutrophil Extracellular Traps. Front. Cell. Infect. Microbiol..

[107] Gabrielli E., Sabbatini S., Roselletti E., Kasper L., Perito S., Hube B., Cassone A., Vecchiarelli A., Pericolini E. (2016). In Vivo Induction of Neutrophil Chemotaxis by Secretory Aspartyl Proteinases of *Candida albicans*. Virulence.

[108] Hornbach A., Heyken A., Schild L., Hube B., Löffler J., Kurzai O. (2009). The Glycosylphosphatidylinositol-Anchored Protease Sap9 Modulates the Interaction of *Candida albicans* with Human Neutrophils. Infect. Immun..

[109] Naglik J.R. (2014). *Candida* Immunity. New J. Sci..

[110] Aoki W., Kitahara N., Miura N., Morisaka H., Yamamoto Y., Kuroda K., Ueda M. (2012). *Candida albicans* Possesses Sap7 as a Pepstatin A-Insensitive Secreted Aspartic Protease. PLoS ONE.

[111] Naglik J.R., Moyes D., Makwana J., Kanzaria P., Tsichlaki E., Weindl G.G., Tappuni A.R., Rodgers C.A., Woodman A.J., Challacombe S.J. (2008). Quantitative Expression of the *Candida albicans* Secreted Aspartyl Proteinase Gene Family in Human Oral and Vaginal Candidiasis. Microbiology.

[112] Whitlock B.B., Gardai S., Fadok V., Bratton D., Henson P.M. (2000). Differential Roles for Alpha(M)Beta(2) Integrin Clustering or Activation in the Control of Apoptosis via Regulation of Akt and ERK Survival Mechanisms. J. Cell Biol..

[113] Behnen M., Leschczyk C., Moller S., Batel T., Klinger M., Solbach W., Laskay T., Möller S., Batel T., Klinger M. (2014). Immobilized Immune Complexes Induce Neutrophil Extracellular Trap Release by Human Neutrophil Granulocytes via Fc RIIIB and Mac-1. J. Immunol..

[114] Zhao S., Shang A., Guo M., Shen L., Han Y., Huang X. (2022). The Advances in the Regulation of Immune Microenvironment by *Candida albicans* and Macrophage Cross-Talk. Front. Microbiol..

[115] Heung L.J. (2020). Monocytes and the Host Response to Fungal Pathogens. Front. Cell. Infect. Microbiol..

[116] Pietrella D., Rachini A., Pandey N., Schild L., Netea M., Bistoni F., Hube B., Vecchiarelli A. (2010). The Inflammatory Response Induced by Aspartic Proteases of *Candida albicans* Is Independent of Proteolytic Activity. Infect. Immun..

[117] Pietrella D., Pandey N., Gabrielli E., Pericolini E., Perito S., Kasper L., Bistoni F., Cassone A., Hube B., Vecchiarelli A. (2013). Secreted Aspartic Proteases of *Candida albicans* Activate the NLRP3 Inflammasome. Eur. J. Immunol..

[118] Gross O., Poeck H., Bscheider M., Dostert C., Hannesschläger N., Endres S., Hartmann G., Tardivel A., Schweighoffer E., Tybulewicz V. (2009). Syk Kinase Signalling Couples to the Nlrp3 Inflammasome for Anti-Fungal Host Defence. Nature.

[119] Hise A.G., Tomalka J., Ganesan S., Patel K., Hall B.A., Brown G.D., Fitzgerald K.A. (2009). An Essential Role for the NLRP3 Inflammasome in Host Defense against the Human Fungal Pathogen *Candida albicans*. Cell Host Microbe.

[120] Gabrielli E., Pericolini E., Luciano E., Sabbatini S., Roselletti E., Perito S., Kasper L., Hube B., Vecchiarelli A. (2015). Induction of Caspase-11 by Aspartyl Proteinases of *Candida albicans* and Implication in Promoting Inflammatory Response. Infect. Immun..

[121] Borg-von Zepelin M., Beggah S., Boggian K., Sanglard D., Monod M. (1998). The Expression of the Secreted Aspartyl Proteinases Sap4 to Sap6 from *Candida albicans* in Murine Macrophages. Mol. Microbiol..

[122] Meiller T.F., Hube B., Schild L., Shirtliff M.E., Scheper M.A., Winkler R., Ton A., Jabra-Rizk M.A. (2009). A Novel Immune Evasion Strategy of *Candida albicans*: Proteolytic Cleavage of a Salivary Antimicrobial Peptide. PLoS ONE.

[123] Bochenska O., Rapala-Kozik M., Wolak N., Aoki W., Ueda M., Kozik A. (2016). The Action of Ten Secreted Aspartic Proteases of Pathogenic Yeast *Candida albicans* on Major Human Salivary Antimicrobial Peptide, Histatin 5. Acta Biochim. Pol..

[124] Ikonomova S.P., Moghaddam-Taaheri P., Jabra-Rizk M.A., Wang Y., Karlsson A.J. (2018). Engineering Improved Variants of the Antifungal Peptide Histatin 5 with Reduced Susceptibility to *Candida albicans* Secreted Aspartic Proteases and Enhanced Antimicrobial Potency. FEBS J..

[125] Ikonomova S.P., Moghaddam-Taaheri P., Wang Y., Doolin M.T., Stroka K.M., Hube B., Karlsson A.J. (2020). Effects of Histatin 5 Modifications on Antifungal Activity and Kinetics of Proteolysis. Protein Sci..

[126] Moghaddam-Taaheri P., Leissa J.A., Eppler H.B., Jewell C.M., Karlsson A.J. (2021). Histatin 5 Variant Reduces *Candida albicans* Biofilm Viability and Inhibits Biofilm Formation. Fungal Genet. Biol..

[127] Vandamme D., Landuyt B., Luyten W., Schoofs L. (2012). A Comprehensive Summary of LL-37, the Factotum Human Cathelicidin Peptide. Cell. Immunol..

[128] Rapala-Kozik M., Bochenska O., Zawrotniak M., Wolak N., Trebacz G., Gogol M., Ostrowska D., Aoki W., Ueda M., Kozik A. (2015). Inactivation of the Antifungal and Immunomodulatory Properties of Human Cathelicidin LL-37 by Aspartic Proteases Produced by the Pathogenic Yeast *Candida albicans*. Infect. Immun..

[129] Frick I.-M., Åkesson P., Herwald H., Mörgelin M., Malmsten M., Nägler D.K., Björck L. (2006). The Contact System—A Novel Branch of Innate Immunity Generating Antibacterial Peptides. EMBO J..

[130] Nordahl E.A., Rydengård V., Mörgelin M., Schmidtchen A. (2005). Domain 5 of High Molecular Weight Kininogen Is Antibacterial. J. Biol. Chem..

[131] Bochenska O., Rapala-Kozik M., Wolak N., Kamysz W., Grzywacz D., Aoki W., Ueda M., Kozik A. (2015). Inactivation of Human Kininogen-Derived Antimicrobial Peptides by Secreted Aspartic Proteases Produced by the Pathogenic Yeast *Candida albicans*. Biol. Chem..

[132] Rothstein D.M., Spacciapoli P., Tran L.T., Xu T., Roberts F.D., Dalla Serra M., Buxton D.K., Oppenheim F.G., Friden P. (2001). Anticandida Activity Is Retained in P-113, a 12-Amino-Acid Fragment of Histatin 5. Antimicrob. Agents Chemother..

[133] Cheng K.-T., Wu C.-L., Yip B.-S., Chih Y.-H., Peng K.-L., Hsu S.-Y., Yu H.-Y., Cheng J.-W. (2020). The Interactions between the Antimicrobial Peptide P-113 and Living *Candida albicans* Cells Shed Light on Mechanisms of Antifungal Activity and Resistance. Int. J. Mol. Sci..

[134] Kalimuthu S., Pudipeddi A., Braś G., Tanner J.A., Rapala-Kozik M., Leung Y.Y., Neelakantan P. (2023). A Heptadeca Amino Acid Peptide Subunit of Cathelicidin LL-37 Has Previously Unreported Antifungal Activity. APMIS.

[135] Germaine G.R., Tellefson L.M. (1981). Effect of pH and Human Saliva on Protease Production by *Candida albicans*. Infect. Immun..

[136] Rüchel R. (1984). A Variety of *Candida* Proteinases and Their Possible Targets of Proteolytic Attack in the Host. Zentralbl Bakteriol. Mikrobiol. Hyg. A.

[137] Gropp K., Schild L., Schindler S., Hube B., Zipfel P.F., Skerka C. (2009). The Yeast *Candida albicans* Evades Human Complement Attack by Secretion of Aspartic Proteases. Mol. Immunol..

[138] Svoboda E., Schneider A.E., Sándor N., Lermann U., Staib P., Kremlitzka M., Bajtay Z., Barz D., Erdei A., Józsi M. (2015). Secreted Aspartic Protease 2 of *Candida albicans* Inactivates Factor H and the Macrophage Factor H-Receptors CR3 (CD11b/CD18) and CR4 (CD11c/CD18). Immunol. Lett..

[139] Valand N., Brunt E., Gazioglu O., Yesilkaya H., Mitchell D., Horley N., Arroo R., Kishore U., Wallis R., Venkatraman Girija U. (2022). Inactivation of the Complement Lectin Pathway by *Candida tropicalis* Secreted Aspartyl Protease-1. Immunobiology.

[140] Hirayasu K., Saito F., Suenaga T., Shida K., Arase N., Oikawa K., Yamaoka T., Murota H., Chibana H., Nakagawa I. (2016). Microbially Cleaved Immunoglobulins Are Sensed by the Innate Immune Receptor LILRA2. Nat. Microbiol..

[141] Rüchel R., Tegeler R., Trost M. (1982). A Comparison of Secretory Proteinases from Different Strains of *Candida albicans*. Med. Mycol..

[142] Rüchel R. (1986). Cleavage of Immunoglobulins by Pathogenic Yeasts of the Genus *Candida*. Microbiol. Sci..

[143] Wich M., Greim S., Ferreira-Gomes M., Krüger T., Kniemeyer O., Brakhage A.A., Jacobsen I.D., Hube B., Jungnickel B. (2021). Functionality of the Human Antibody Response to *Candida albicans*. Virulence.

[144] Guevara-Lora I., Bras G., Karkowska-Kuleta J., González-González M., Ceballos K., Sidlo W., Rapala-Kozik M. (2020). Plant-Derived Substances in the Fight Against Infections Caused by *Candida* Species. Int. J. Mol. Sci..

[145] Kumar R., Shukla P.K. (2010). Amphotericin B Resistance Leads to Enhanced Proteinase and Phospholipase Activity and Reduced Germ Tube Formation in *Candida albicans*. Fungal Biol..

[146] Feng W., Yang J., Pan Y., Xi Z., Qiao Z., Ma Y. (2016). The Correlation of Virulence, Pathogenicity, and Itraconazole Resistance with SAP Activity in *Candida albicans* Strains. Can. J. Microbiol..

[147] Kadry A.A., El-Ganiny A.M., El-Baz A.M. (2018). Relationship between Sap Prevalence and Biofilm Formation among Resistant Clinical Isolates of *Candida albicans*. Afr. Health Sci..

[148] Gerges M.A., Fahmy Y.A., Hosny T., Gandor N.H., Mohammed S.Y., Mohamed T.M.A., Abdelmoteleb N.E.M., Esmaeel N.E. (2023). Biofilm Formation and Aspartyl Proteinase Activity and Their Association with Azole Resistance Among *Candida albicans* Causing Vulvovaginal Candidiasis, Egypt. Infect. Drug Resist..

[149] Wu T., Wright K., Hurst S.F., Morrison C.J. (2000). Enhanced Extracellular Production of Aspartyl Proteinase, a Virulence Factor, by *Candida albicans* Isolates Following Growth in Subinhibitory Concentrations of Fluconazole. Antimicrob. Agents Chemother..

[150] Copping V.M.S., Barelle C.J., Hube B., Gow N.A.R., Brown A.J.P., Odds F.C. (2005). Exposure of *Candida albicans* to Antifungal Agents Affects Expression of SAP2 and SAP9 Secreted Proteinase Genes. J. Antimicrob. Chemother..

[151] Barelle C.J., Duncan V.M.S., Brown A.J.P., Gow N.A.R., Odds F.C. (2008). Azole Antifungals Induce Up-Regulation of *SAP4*, *SAP5* and *SAP6* Secreted Proteinase Genes in Filamentous *Candida albicans* Cells In Vitro and In Vivo. J. Antimicrob. Chemother..

[152] El-Houssaini H.H., Elnabawy O.M., Nasser H.A., Elkhatib W.F. (2019). Correlation between Antifungal Resistance and Virulence Factors in *Candida albicans* Recovered from Vaginal Specimens. Microb. Pathog..

[153] Figueiredo-Carvalho M.H.G., Ramos L.D.S., Barbedo L.S., Oliveira J.C.A.D., Santos A.L.S.D., Almeida-Paes R., Zancopé-Oliveira R.M. (2017). Relationship between the Antifungal Susceptibility Profile and the Production of Virulence-Related Hydrolytic Enzymes in Brazilian Clinical Strains of *Candida glabrata*. Mediat. Inflamm..

[154] Askari F., Rasheed M., Kaur R. (2022). The Yapsin Family of Aspartyl Proteases Regulate Glucose Homeostasis in *Candida glabrata*. J. Biol. Chem..

[155] Miyazaki T., Izumikawa K., Yamauchi S., Inamine T., Nagayoshi Y., Saijo T., Seki M., Kakeya H., Yamamoto Y., Yanagihara K. (2011). The Glycosylphosphatidylinositol-Linked Aspartyl Protease Yps1 Is Transcriptionally Regulated by the Calcineurin-Crz1 and Slt2 MAPK Pathways in *Candida glabrata*: Transcriptional Regulation of YPS1 in C. Glabrata. FEMS Yeast Res..

[156] Bairwa G., Rasheed M., Taigwal R., Sahoo R., Kaur R. (2014). GPI (Glycosylphosphatidylinositol)-Linked Aspartyl Proteases Regulate Vacuole Homoeostasis in *Candida glabrata*. Biochem. J..

[157] Bairwa G., Kaur R. (2011). A Novel Role for a Glycosylphosphatidylinositol-anchored Aspartyl Protease, CgYps1, in the Regulation of pH Homeostasis in *Candida glabrata*. Mol. Microbiol..

[158] Morrison C.J., Hurst S.F., Reiss E. (2003). Competitive Binding Inhibition Enzyme-Linked Immunosorbent Assay That Uses the Secreted Aspartyl Proteinase of *Candida albicans* as an Antigenic Marker for Diagnosis of Disseminated Candidiasis. Clin. Diagn. Lab. Immunol..

[159] Mavor A., Thewes S., Hube B. (2005). Systemic Fungal Infections Caused by *Candida* Species: Epidemiology, Infection Process and Virulence Attributes. CDT.

[160] Wang L., Wang Y., Gao X., Zhi Gang J., Liu J., Dong S. (2013). Detection of *Candida albicans* Sap2 in Cancer Patient Serum Samples by an Indirect Competitive Enzyme-Linked Immunosorbent Assay for the Diagnosis of Candidiasis. Indian J. Pathol. Microbiol..

[161] De Bernardis F., Agatensi L., Ross I.K., Emerson G.W., Lorenzini R., Sullivan P.A., Cassone A. (1990). Evidence for a Role for Secreted Aspartate Proteinase of *Candida albicans* in Vulvovaginal Candidiasis. J. Infect. Dis..

[162] Louie A., Dixon D.M., el-Maghrabi E.A., Burnett J.W., Baltch A.L., Smith R.P. (1994). Relationship between *Candida albicans* Epidermolytic Proteinase Activity and Virulence in Mice. J. Med. Vet. Mycol..

[163] Schaller M., Schackert C., Korting H.C., Januschke E., Hube B. (2000). Invasion of *Candida albicans* Correlates with Expression of Secreted Aspartic Proteinases during Experimental Infection of Human Epidermis. J. Investig. Dermatol..

[164] Rüchel R., Böning-Stutzer B., Mari A. (1988). A Synoptical Approach to the Diagnosis of Candidosis, Relying on Serological Antigen and Antibody Tests, on Culture, and on Evaluation of Clinical Data. Mycoses.

[165] Na B.-K., Song C.-Y. (1999). Use of Monoclonal Antibody in Diagnosis of Candidiasis Caused by *Candida albicans*: Detection of Circulating Aspartyl Proteinase Antigen. Clin. Diagn. Lab. Immunol..

[166] Aoki W., Kitahara N., Fujita A., Shibasaki S., Morisaka H., Kuroda K., Ueda M. (2013). Detection of *Candida albicans* by Using a Designed Fluorescence-Quenched Peptide. J. Biosci. Bioeng..

[167] Gheit M.I., Mohamed T.M., Abdelwahab M.A. (2023). Evaluation of Polyclonal Antiserum Against Secretory Aspartyl Proteinase of *Candida albicans* as a Potential Serodiagnostic Tool for Invasive Candidiasis. Turk. J. Immunol..

[168] Shukla M., Chandley P., Kaur H., Ghosh A.K., Rudramurthy S.M., Rohatgi S. (2021). Expression and Purification along with Evaluation of Serological Response and Diagnostic Potential of Recombinant Sap2 Protein from *C. parapsilosis* for Use in Systemic Candidiasis. J. Fungi.

[169] Cassone A., Boccanera M., Adriani D., Santoni G., De Bernardis F. (1995). Rats Clearing a Vaginal Infection by *Candida albicans* Acquire Specific, Antibody-Mediated Resistance to Vaginal Reinfection. Infect. Immun..

[170] De Bernardis F., Boccanera M., Adriani D., Spreghini E., Santoni G., Cassone A. (1997). Protective Role of Antimannan and Anti-Aspartyl Proteinase Antibodies in an Experimental Model of *Candida albicans* Vaginitis in Rats. Infect. Immun..

[171] De Bernardis F., Boccanera M., Adriani D., Girolamo A., Cassone A. (2002). Intravaginal and Intranasal Immunizations Are Equally Effective in Inducing Vaginal Antibodies and Conferring Protection against Vaginal Candidiasis. Infect. Immun..

[172] Sandini S., La Valle R., Deaglio S., Malavasi F., Cassone A., De Bernardis F. (2011). A Highly Immunogenic Recombinant and Truncated Protein of the Secreted Aspartic Proteases Family (rSap2t) of *Candida albicans* as a Mucosal Anticandidal Vaccine. FEMS Immunol. Med. Microbiol..

[173] De Bernardis F., Amacker M., Arancia S., Sandini S., Gremion C., Zurbriggen R., Moser C., Cassone A. (2012). A Virosomal Vaccine against Candidal Vaginitis: Immunogenicity, Efficacy and Safety Profile in Animal Models. Vaccine.

[174] Moser C., Amacker M., Zurbriggen R. (2011). Influenza Virosomes as a Vaccine Adjuvant and Carrier System. Expert Rev. Vaccines.

[175] Mellid-Carballal R., Gutierrez-Gutierrez S., Rivas C., Garcia-Fuentes M. (2023). Viral Protein-Based Nanoparticles (Part 2): Pharmaceutical Applications. Eur. J. Pharm. Sci..

[176] Akhtar N., Singh A., Upadhyay A.K., Mannan M.A. (2022). Design of a Multi-Epitope Vaccine against the Pathogenic Fungi *Candida tropicalis* Using an in Silico Approach. J. Genet. Eng. Biotechnol..

[177] Akhtar N., Magdaleno J.S.L., Ranjan S., Wani A.K., Grewal R.K., Oliva R., Shaikh A.R., Cavallo L., Chawla M. (2023). Secreted Aspartyl Proteinases Targeted Multi-Epitope Vaccine Design for *Candida dubliniensis* Using Immunoinformatics. Vaccines.

[178] Shukla M., Rohatgi S. (2020). Vaccination with Secreted Aspartyl Proteinase 2 Protein from *Candida parapsilosis* Can Enhance Survival of Mice during *C. tropicalis* -Mediated Systemic Candidiasis. Infect. Immun..

[179] Wu T., Samaranayake L.P., Leung W.K., Sullivan P.A. (1999). Inhibition of Growth and Secreted Aspartyl Proteinase Production in *Candida albicans* by Lysozyme. J. Med. Microbiol..

[180] Wagener J., Schneider J.J., Baxmann S., Kalbacher H., Borelli C., Nuding S., Küchler R., Wehkamp J., Kaeser M.D., Mailänder-Sanchez D. (2013). A Peptide Derived from the Highly Conserved Protein GAPDH Is Involved in Tissue Protection by Different Antifungal Strategies and Epithelial Immunomodulation. J. Investig. Dermatol..

[181] Rüchel R., Ritter B., Schaffrinski M. (1990). Modulation of Experimental Systemic Murine Candidosis by Intravenous Pepstatin. Zentralblatt Bakteriol..

[182] Backman D., Danielson U.H. (2003). Kinetic and Mechanistic Analysis of the Association and Dissociation of Inhibitors Interacting with Secreted Aspartic Acid Proteases 1 and 2 from *Candida albicans*. Biochim. Biophys. Acta (BBA)-Proteins Proteom..

[183] Cadicamo C.D., Mortier J., Wolber G., Hell M., Heinrich I.E., Michel D., Semlin L., Berger U., Korting H.C., Höltje H.-D. (2013). Design, Synthesis, Inhibition Studies, and Molecular Modeling of Pepstatin Analogues Addressing Different Secreted Aspartic Proteinases of *Candida albicans*. Biochem. Pharmacol..

[184] Dostál J., Brynda J., Hrušková-Heidingsfeldová O., Sieglová I., Pichová I., Řezáčová P. (2009). The Crystal Structure of the Secreted Aspartic Protease 1 from *Candida parapsilosis* in Complex with Pepstatin A. J. Struct. Biol..

[185] Dostál J., Brynda J., Vaňková L., Zia S.R., Pichová I., Heidingsfeld O., Lepšík M. (2021). Structural Determinants for Subnanomolar Inhibition of the Secreted Aspartic Protease Sapp1p from *Candida Parapsilosis*. J. Enzym. Inhib. Med. Chem..

[186] Gruber A., Berlit J., Speth C., Lass-Flörl C., Kofler G., Nagl M., Borg-von Zepelin M., Dierich M.P., Würzner R. (1999). Dissimilar Attenuation of *Candida albicans* Virulence Properties by Human Immunodeficiency Virus Type 1 Protease Inhibitors. Immunobiology.

[187] Gruber A., Speth C., Lukasser-Vogl E., Zangerle R., Borg-von Zepelin M., Dierich M.P., Würzner R. (1999). Human Immunodeficiency Virus Type 1 Protease Inhibitor Attenuates *Candida albicans* Virulence Properties in Vitro. Immunopharmacology.

[188] Korting H.C., Schaller M., Eder G., Hamm G., Böhmer U., Hube B. (1999). Effects of the Human Immunodeficiency Virus (HIV) Proteinase Inhibitors Saquinavir and Indinavir on In Vitro Activities of Secreted Aspartyl Proteinases of *Candida albicans* Isolates from HIV-Infected Patients. Antimicrob. Agents Chemother..

[189] Cassone A., De Bernardis F., Torosantucci A., Tacconelli E., Tumbarello M., Cauda R. (1999). In Vitro and In Vivo Anticandidal Activity of Human Immunodeficiency Virus Protease Inhibitors. J. Infect. Dis..

[190] Borg-von Zepelin M., Meyer I., Thomssen R., Würzner R., Sanglard D., Telenti A., Monod M. (1999). HIV-Protease Inhibitors Reduce Cell Adherence of *Candida albicans* Strains by Inhibition of Yeast Secreted Aspartic Proteases. J. Investig. Dermatol..

[191] Bektić J., Lell C.P., Fuchs A., Stoiber H., Speth C., Lass-Flörl C., Borg-von Zepelin M., Dierich M.P., Würzner R. (2001). HIV Protease Inhibitors Attenuate Adherence of *Candida albicans* to Epithelial Cells in Vitro. FEMS Immunol. Med. Microbiol..

[192] Blanco M.T., Hurtado C., Pérez-giraldo C., Morán F.J., González-velasco C., Gómezgarcía A.C. (2003). Effect of Ritonavir and Saquinavir on *Candida albicans* Growth Rate and In Vitro Activity of Aspartyl Proteinases. Med. Mycol..

[193] Schaller M., Bein M., Korting H.C., Baur S., Hamm G., Monod M., Beinhauer S., Hube B. (2003). The Secreted Aspartyl Proteinases Sap1 and Sap2 Cause Tissue Damage in an In Vitro Model of Vaginal Candidiasis Based on Reconstituted Human Vaginal Epithelium. Infect. Immun..

[194] Santos A.L.S., Braga-Silva L.A., Gonçalves D.S., Ramos L.S., Oliveira S.S.C., Souza L.O.P., Oliveira V.S., Lins R.D., Pinto M.R., Muñoz J.E. (2021). Repositioning Lopinavir, an HIV Protease Inhibitor, as a Promising Antifungal Drug: Lessons Learned from *Candida albicans*—In Silico, In Vitro and In Vivo Approaches. J. Fungi.

[195] Asencio M.A., Garduño E., Pérez-Giraldo C., Blanco M.T., Hurtado C., Gómez-García A.C. (2005). Exposure to Therapeutic Concentrations of Ritonavir, but Not Saquinavir, Reduces Secreted Aspartyl Proteinase of *Candida parapsilosis*. Chemotherapy.

[196] Zhang Z., ElSohly H.N., Jacob M.R., Pasco D.S., Walker L.A., Clark A.M. (2002). Natural Products Inhibiting *Candida albicans* Secreted Aspartic Proteases from *Lycopodium Cernuum*. J. Nat. Prod..

[197] Höfling J.F., Mardegan R.C., Anibal P.C., Furletti V.F., Foglio M.A. (2011). Evaluation of Antifungal Activity of Medicinal Plant Extracts Against Oral *Candida albicans* and Proteinases. Mycopathologia.

[198] Liu N., Zhang N., Zhang S., Zhang L., Liu Q. (2021). Phloretin Inhibited the Pathogenicity and Virulence Factors against *Candida albicans*. Bioengineered.

[199] Christopeit T., Øverbø K., Danielson U., Nilsen I. (2013). Efficient Screening of Marine Extracts for Protease Inhibitors by Combining FRET Based Activity Assays and Surface Plasmon Resonance Spectroscopy Based Binding Assays. Mar. Drugs.

[200] Gu W., Guo D., Zhang L., Xu D., Sun S. (2016). The Synergistic Effect of Azoles and Fluoxetine against Resistant *Candida albicans* Strains Is Attributed to Attenuating Fungal Virulence. Antimicrob. Agents Chemother..

[201] Falkensammer B., Pleyer L., Ressler S., Berg A., Borg-von Zepelin M., Nagl M., Lass-Flörl C., Speth C., Dierich M.P., Würzner R. (2008). Basidiomycete Metabolites Attenuate Virulence Properties of *Candida albicans in Vitro*. Mycoses.

[202] Jebali A., Hajjar F.H.E., Hekmatimoghaddam S., Kazemi B., De La Fuente J.M., Rashidi M. (2014). Triangular Gold Nanoparticles Conjugated with Peptide Ligands: A New Class of Inhibitor for *Candida albicans* Secreted Aspartyl Proteinase. Biochem. Pharmacol..

[203] Goldman R.C., Frost D.J., Capobianco J.O., Kadam S., Rasmussen R.R., Abad-Zapatero C. (1995). Antifungal Drug Targets: *Candida* Secreted Aspartyl Protease and Fungal Wall Beta-Glucan Synthesis. Infect. Agents Dis..

[204] Abad-Zapatero C., Goldman R., Muchmore S.W., Hutchins C., Stewart K., Navaza J., Payne C.D., Ray T.L. (1996). Structure of a Secreted Aspartic Protease from *C. albicans* Complexed with a Potent Inhibitor: Implications for the Design of Antifungal Agents. Protein Sci..

[205] Stewart K., Abad-Zapatero C. (2001). Candida Proteases and Their Inhibition Prospects for Antifungal Therapy. Curr. Med. Chem..

[206] Pranav Kumar S.K., Kulkarni V.M. (2002). Insights into the Selective Inhibition of *Candida albicans* Secreted Aspartyl Protease: A Docking Analysis Study. Bioorganic Med. Chem..

[207] Kathwate G.H., Karuppayil S.M. (2013). Antifungal Properties of the Anti-Hypertensive Drug: Aliskiren. Arch. Oral Biol..

[208] Gholam G.M., Firdausy I.A. (2022). Molecular Docking Study of Natural Compounds from Red Betel (Piper Crocatum Ruiz & Pav) as Inhibitor of Secreted Aspartic Proteinase 5 (Sap 5) in *Candida albicans*. Sasambo J. Pharm..

